# Identifying social factors amongst older individuals in linked electronic health records: An assessment in a population based study

**DOI:** 10.1371/journal.pone.0189038

**Published:** 2017-11-30

**Authors:** Anu Jain, Albert J. van Hoek, Jemma L. Walker, Rohini Mathur, Liam Smeeth, Sara L. Thomas

**Affiliations:** 1 Faculty of Epidemiology and Population Health, London School of Hygiene and Tropical Medicine, London, United Kingdom; 2 Statistics, Modelling and Economics Department, Public Health England, London, United Kingdom; Istituto Di Ricerche Farmacologiche Mario Negri, ITALY

## Abstract

Identification and quantification of health inequities amongst specific social groups is a pre-requisite for designing targeted healthcare interventions. This study investigated the recording of social factors in linked electronic health records (EHR) of individuals aged ≥65 years, to assess the potential of these data to identify the social determinants of disease burden and uptake of healthcare interventions. Methodology was developed for ascertaining social factors recorded on or before a pre-specified index date (01/01/2013) using primary care data from Clinical Practice Research Datalink (CPRD) linked to hospitalisation and deprivation data in a cross-sectional study. Social factors included: religion, ethnicity, immigration status, small area-level deprivation, place of residence (including communal establishments such as care homes), marital status and living arrangements (e.g. living alone, cohabitation). Each social factor was examined for: completeness of recording including improvements in completeness by using other linked EHR, timeliness of recording for factors that might change over time and their representativeness (compared with English 2011 Census data when available). Data for 591,037 individuals from 389 practices from England were analysed. The completeness of recording varied from 1.6% for immigration status to ~80% for ethnicity. Linkages provided the deprivation data (available for 82% individuals) and improved completeness of ethnicity recording from 55% to 79% (when hospitalisation data were added). Data for ethnicity, deprivation, living arrangements and care home residence were comparable to the Census data. For time-varying variables such as residence and living alone, ~60% and ~35% respectively of those with available data, had this information recorded within the last 5 years of the index date. This work provides methods to identify social factors in EHR relevant to older individuals and shows that factors such as ethnicity, deprivation, not living alone, cohabitation and care home residence can be ascertained using these data. Applying these methodologies to routinely collected data could improve surveillance programmes and allow assessment of health equity in specific healthcare studies.

## Introduction

Health inequity is defined as unjust differences in health status amongst different social groups, and may be explained by the distribution of social determinants of health.[1] Health inequities not only exist between countries, but are apparent within a country.[2] In the UK, reducing health inequities is a statutory requirement and is a common theme in the area of health improvement in the Public Health Outcome Framework.[3–6] In order to attain health equity, it is vital that the disadvantaged individuals are identified to quantify the problem and formulate targeted public health interventions. Increase in life expectancy has led to an aging population, and globally the proportion of individuals aged ≥60 years is projected to nearly double by 2050 from ~12% in 2013.[7, 8]The higher prevalence of chronic diseases in this age group is associated with greater disability and requirement for long-term care, necessitating changes in health and social care delivery.[9, 10] The effect of ageing on future health expenditure will depend on health expectancy: a measure that takes both life expectancy and disability into account.[11, 12] A 2015 systematic review reported associations of social factors such as gender, ethnicity, and socioeconomic position (including education) with inequalities in healthy life expectancy amongst older individuals.[13] Similarly, amongst individuals aged 50–65 years, social class, education, wealth and income were found to be associated with all three indicators of health expectancies: disability-free, illness-free and healthy life expectancy.[9] Living alone is also known to be associated with higher morbidity and mortality.[14] Uptake of preventative measures such as vaccination amongst older individuals has been shown to be lower amongst immigrants, individuals of certain ethnicities, and those living alone.[15, 16]

One of the recommendations by the World Health Organisation’s (WHO) Commission on Social Determinants of Health (CSDH) in 2008 was setting of global and national equity surveillance systems to monitor health inequities routinely.[17] Surveillance programmes in the UK lack detailed information about social factors.[18] However, these factors potentially could be ascertained using routinely collected electronic health records (EHR). This provides an opportunity to utilise routinely collected data to improve surveillance programmes and to assess health inequities in specific studies.

The Clinical Practice Research Datalink (CPRD) is the world’s largest primary care database, comprising anonymised patient information from ~7% of the UK population and including >79 million person-years of follow-up cumulatively.[19, 20] These EHR comprise not only data relating to primary care consultations, but also records of referrals to and feedback from secondary care.[21] Data in CPRD are representative of the UK population and are quality assured at both patient and general practice level.[20, 21] In England, linkage of the CPRD data at the individual level (from ~75% of English practices that consent to linkages) is available for hospitalisation data (Hospital Episode Statistics, HES)[22] and deprivation data (e.g. quintiles of Index of Multiple deprivation (IMD) score).[21, 23] For deprivation data, the linkage is made at the lower layer super output areas (LSOA) level, which covers a population of 1000–3000.[23] The completeness and quality of recording of one social factor in the CPRD, namely ethnicity, have been assessed by Mathur *et al* using data up to 2012 and focusing chiefly on the time during which GPs were financially incentivised to record the ethnicity of newly registered patients.[24] This study showed that in linked CPRD-HES data, completeness of recording reached 90% in newly registered patients. However, this analysis did not include assessment of recording specifically for older patients in CPRD, and was not extended to examine completeness after incentivisation was withdrawn in 2011.[25] In the UK, EHR have also been utilised to study cohabitation and care home residence,[26–28] but these studies did not provide information on timeliness or representativeness of recording of these factors and did not utilise linked hospitalisation data. To our knowledge, simultaneous investigation of the quality and completeness of recording in CPRD of the social determinants of disease burden or healthcare usage in older populations have not yet been undertaken.

This study aimed to investigate the utility of the CPRD and linked databases in ascertaining social factors that are potential determinants of disease burden and inequitable healthcare interventions targeted towards older individuals, to discuss challenges associated with using routinely collected data and to supplement and enhance existing surveillance methods with the overarching goal of informing interventions to reduce health inequities.

## Methods

### Data source and study date

This was a cross-sectional study using CPRD data linked to HES data and deprivation data (IMD 2010) in England. It investigated the historical recording of social factors among individuals aged ≥65 years, actively registered with a CPRD practice on a randomly chosen index date (1st January 2013), to allow assessment of both completeness and timeliness of recording of social factor data. Active registration on 01/01/2013 was determined by ensuring that patients’ start dates (the later of their registration date with the practice or the date the practice reached CPRD-defined quality criteria[21]) fell before the index date and their end dates (the earliest of their transfer out date, date of death or practice last collection date) were after the index date.

CPRD data are supplied in ten different files,[19] of which eight (patient, practice, clinical, consultation, additional clinical details, immunisation, referral and test files) were used for this study (S1 Table). These files include information about patients’ demography, lifestyle factors, clinical details, feedback from secondary care, therapy and laboratory results, stored in form of medical, therapy and other codes used by the GP practice staff.[21]

### Social factors examined

In this study, social factors relevant at an individual level and informed by the conceptual framework of the WHO’s CSDH,[1] were examined in CPRD and included: religion, ethnicity, immigration status, deprivation based on LSOA of each individual’s residence,[23] living arrangements (living alone and cohabitation), residence (place of residence and homelessness) and marital status.

Lists of medical codes (S2 Table) for each factor were compiled by searching the CPRD’s Read code dictionary [21] for specific and broader text terms (using wild card searches) encompassing all social factors of interest. This was an iterative process that subsequently included a hierarchical search of the Read codes identified. The number of codes identified for these factors ranged from 86–465 (S2 Table). Further information (S1 Table) was accrued from other sources within the dataset as follows: the consultation files provided codes (‘consultation type’) on where the consultation took place and thus patients’ residence (for example in a care home), while the patient files provided information regarding patients’ marital status and their family number.[29] The latter variable can identify individuals sharing the same household and therefore can be used to get information for living arrangements (living alone, cohabitation), marital status and care home status. Similarly, the additional clinical details files provided coded information (‘entity type’) about residence, living alone and marital status. The linked hospitalisation data from HES provided additional information for ethnicity and residence, whilst the deprivation data provided deprivation scores for individuals’ LSOA as IMD quintile. The multiple code lists thus generated were discussed amongst the three of the authors (AJ, SLT and AJvH). These code lists were then utilised to systematically search for the Read codes in the clinical, immunisation, referral and test files. Additional information was sought from consultation type and entity types in the consultation and additional clinical details files respectively and also from the patient file and from linked HES and deprivation data. Some factors also provided information about another social factors: for example an individual coded as living alone was deemed not to be cohabiting, whereas an individual residing in a care home was considered not to be living alone.

The following example illustrates how information for social factors was assimilated. Type of residence (whether a patient lived in their own home, in sheltered accommodation, or in a care home) can be recorded in numerous way in both CPRD and HES. In CPRD, this information can be determined using the medical codes within multiple files as described above, using the entity type 132 for residence in the additional clinical details file, from the consultation file (e.g. “nursing-home visit”) and from the family number (as described below); residence data are also potentially available in HES by using information about individual’s location prior to hospital admission.

### Exposure variables definition and categorisation

The code lists for the social factors of interest (religion, ethnicity, living arrangements (including living alone and cohabitation), immigration status, deprivation, residence (including place of residence and homelessness) and marital status) are presented in S2 Table. Ethnicity codes were those recommended for use by the Quality and Outcomes Framework, as used by Mathur *et al*.[24] Family number was used to derive additional information by modifying approaches used in previous studies,[27, 28, 30] as follows. Two adults, living in a household size of two or three, were identified as cohabiting (adults living in a couple) if the age difference between the couple was ≤15 years and age difference between the other household occupants and those living in a couple was >15 years. Couples identified as cohabiting were also allocated `partner-uncategorised’ category for marital status. Individuals from household size of two or more were identified as not living alone. Based on previous studies [26, 31, 32] care home was defined as a household with >3 individuals aged ≥65 years and if their total count was more than individuals aged <65 years. In sensitivity analyses households with >3 individuals aged ≥65 years and ≤3 individual aged ≤50 years were defined as a care home.

Religion was categorised into eight categories (Buddhists, Christians, Hindus, Jews, Muslims, Sikhs, Others and no religion (atheists)) to ensure comparability with Census data.[33] We hypothesised that certain minority religions might be more likely to be coded by GPs, and explored this by categorising one religion (Muslim) as a binary (yes/no) variable. Ethnicity was categorised in five groups: White, South Asian, Black, Others and Mixed as per the UK 2011 Census.[34]

Living alone and cohabitation were coded as binary variables (yes/no). Immigration status, a binary variable (immigrant/ not immigrant) was defined using: i) country of birth information and (to increase completeness of ascertainment) ii) codes for the first language spoken (S2 Table).

Place of residence had four categories: living in a care home, sheltered accommodation, other places of residence (e.g. prison, hospice, hostel, welfare home) and living in a household. Care home status was also considered as a binary (yes/no) variable, on the assumption that being in a care home might be more completely recorded by GPs than other places of residence (e.g living in a household). Homelessness was also a binary variable.

Relationship status was characterised by using following seven categories: single, married/civil partnership, widow/er, divorced, separated, partner-other (e.g. common-law husband/wife) and partner (uncategorised). As the last category was non-specific, an algorithm was developed to obtain more specific marital status information. If the ‘partner uncategorised’ status was preceded by any of the following three categories: 1) Single/engaged 2) Married/ civil partnership and 3) Partner-other category, the ‘partner uncategorised’ category was updated to that of the earlier observation.

Deprivation status is a composite score of 38 indicators for seven domains of deprivation (income, health and disability, employment, education and training, housing, living environment and crime).[23] These indices are available at the small area level (LSOA) as quintiles: quintile one representing the least deprived to quintile five representing the most deprived.[23]

### Analysis

For the purposes of recording, the social factors that were likely to change with time (e.g. marital status, living alone status) were treated as time-varying exposure variables whereas ethnicity, religion and immigration status were deemed to be time-invariant.

In CPRD the event date (the date the event occurred as recorded by the GP) was used to ascertain when the factor was recorded in relation to the index date. If the event date was missing then the system date (the date when the event was recorded on the GP system) was used for these observations.[19] For information extracted from the patient files (such as marital status, family number), which does not include event dates, a conservative estimate of the date of recording was taken, using the date the patient registered with the practice[19], and the hospital admission date was utilised for HES data.[22]

All mentions of each factor of interest were identified within a patient’s linked records. Observations providing discordant information for a factor on the same date for a patient were excluded and the social factor recorded nearest the index date was used.

As family number provided information for social factors indirectly, and the date of recording this variable was unclear, information from family number was used only when data for a particular social factor were unavailable from other sources in CPRD or HES for that patient.

For each social factor, the following information was analysed:

#### (a) Completeness of recording and contribution from linkages

Completeness was described as the percentage of total patients who had data available: i) within CPRD and ii) within CPRD linked to HES, to investigate the extent to which use of the linked data increased completeness. For time-varying variables, completeness was determined in the period before or on the index date (taking the value nearest the index date). However, for time-invariant variables such as country of birth, ethnicity and religion, completeness of recording included both the period before and after the index date. For ethnicity, we further investigated completeness of recording by GPs over time by plotting completeness against year of registration with the general practice. We also assessed the contribution of family number by looking at completeness with and without family number data.

#### (b) Representativeness

The representativeness of the recorded data was investigated by comparing the distribution of each social factor amongst those with non-missing data with the distribution recorded in the 2011 Census (data from England for individuals aged ≥65 years). When applicable, we also considered the binary version of multi-category variables (i.e. care home status instead of the four-category variable for residence, and Muslim religion). For all binary variables, (immigration status, care home status, Muslim religion, homelessness, living alone, and cohabitation status) we assessed representativeness assuming that those without a code did not have the attribute, and thus compared the distribution of each factor among the entire study population to the Census data.

#### (c) Timeliness

For the time-varying factors, the duration between index date and the record nearest to the index date was calculated. Factors recorded more than five years before the index date were not considered timely.

## Ethics approval

All data were anonymised prior to receipt by the authors. Approval for this study was obtained from the Independent Scientific Advisory Committee of the Medicines and Healthcare products Regulatory Agency (Ref: 15_253) and the Research Ethics Committee of the London School of Hygiene and Tropical Medicine (reference:10524). The original Independent Scientific Advisory Committee protocol was made available to the reviewers of this paper.

Data were analysed using Stata-14 software (StataCorp, College Station, TX, USA).

## Results

The study population comprised 591,037 patients from 389 GP practices in England. More than half of the study participants (55%) were females, and 53% were aged between 65–74 years at the index date, with ~14% aged ≥85 years. The median age for women was 75.5 years (interquartile range (IQR): 69.5–82.5 years) whilst for men it was 73.5 years (IQR: 68.5–79.5 years). Information for one or more time-invariant social factors was available for~92% (n = 541,197) of the study population, while 75% (n = 444,827) had data for one or more time-varying social factors. Overall, ~98% (n = 578,410) had information for one or more social factors. Further details of the overall pattern of completeness is given in S3 Table; only 45 patients (<0.01%) had data for all seven social factors included in this study while ~21% (n = 123,450) had information for three social factors: ethnicity, IMD and living alone. The system date was used to replace missing event date for only 0.4% (n = 2,219) of the study population (S4 Table). The maximum number (n = 456; <0.1%) of patients were excluded due to discordant information recorded on the same date (S4 Table) were for the factor: living alone.

### Completeness of recording for individual social factors, and contribution from linkages

**Fig 1:**
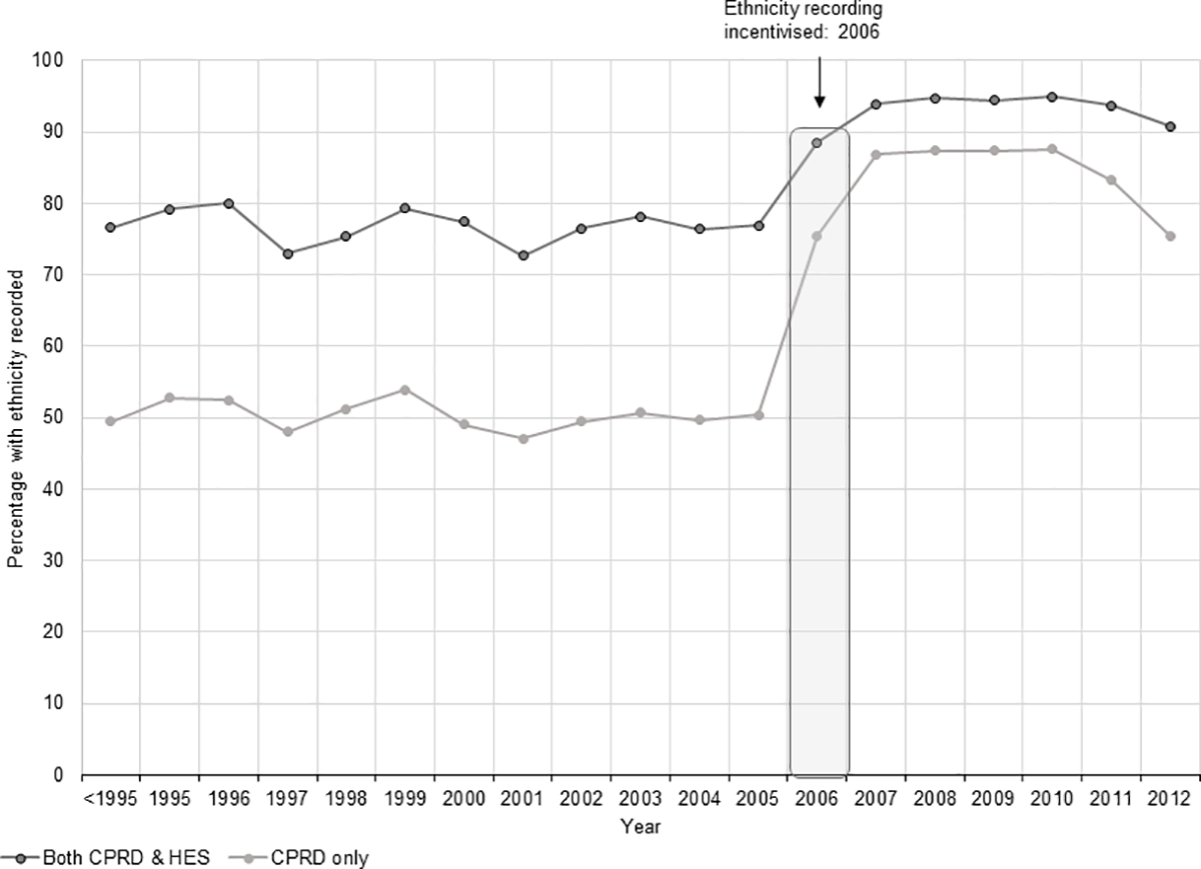
Patients with ethnicity records in Clinical Practice Research Datalink and Hospital Episode Statistics over time. Abbreviations: CPRD Clinical Practice Research Datalink HES Hospital Episode Statistics.

Completeness of recording for all social factors was better for females and amongst the oldest individuals (aged ≥85 years) for all factors except for religion, immigration status and IMD score (Table 1). Of the seven social factors ascertained, recording for deprivation data and ethnicity were the most complete, at ~82% (n = 486,426) and ~80% (n = 469,557) respectively (Table 1). The recording of ethnicity over time showed an increase in completeness in the year 2006 (when incentivisation was introduced) with a slight downward trend in 2011 and 2012 (Fig 1).

**Table 1:** Proportion of individuals with information on social factors available in Clinical Practice Research Datalink and Hospital Episodes Statistics: Age, sex and database distribution (N = 591037).

Study population stratified (N)	Codes available for social factors in CPRD and linked data: proportion* (95% confidence interval)								
	Marital status codes	Living arrangements		Residence		Religion codes	Ethnicity codes	IMD	Immigration status codes
		Cohabitation (yes/no) codes	Living alone (yes/no) codes	Place of residence codes	Homeless (yes/no) codes				
**Age groups in years (N)**									
65–69 (183382)	26.10%	21.50%	26.60%	4.60%	4.60%	2.40%	76.90%	82.50%	1.60%
	(25.9–26.3)	(21.4–21.7)	(26.4–26.8)	(4.5–4.6)	(4.5–4.7)	(2.3–2.5)	(76.7–77.1)	(82.3–82.7)	(1.5–1.7)
70–74 (131552)	25.90%	21%	26.60%	5.70%	5.70%	2.70%	80%	82.60%	1.80%
	(25.7–26.2)	(20.8–21.2)	(26.4–26.9)	(5.6–5.9)	(5.6–5.9)	(2.6–2.8)	(79.8–80.2)	(82.4–82.8)	(1.8–1.9)
75–79 (109628)	27.10%	21.40%	27.80%	8.20%	8.20%	2.90%	80.10%	82.30%	1.80%
	(26.8–27.3)	(21.1–21.6)	(27.5–28)	(8.1–8.4)	(8.1–8.4)	(2.8–3)	(79.8–80.3)	(82–82.5)	(1.7–1.9)
80–84 (84473)	28.70%	21.80%	30.30%	13.10%	13.20%	2.70%	81.10%	82%	1.60%
	(28.4–29)	(21.5–22)	(30–30.6)	(12.9–13.4)	(12.9–13.4)	(2.5–2.8)	(80.9–81.4)	(81.8–82.3)	(1.5–1.7)
85–89 (51278)	30.80%	24.30%	37.70%	25.20%	25.30%	2.60%	82.40%	82%	1.40%
	(30.4–31.2)	(23.9–24.6)	(37.3–38.1)	(24.8–25.6)	(24.9–25.7)	(2.5–2.8)	(82.1–82.7)	(81.6–82.3)	(1.3–1.5)
≥90 (30724)	29.50%	23.40%	43.90%	38.20%	38.30%	2.50%	80.70%	81.60%	0.97%
	(29–30)	(22.9–23.9)	(43.3–44.4)	(37.7–38.7)	(37.8–38.9)	(2.3–2.7)	(80.2–81.1)	(81.1–82)	(0.9–1.1)
**Gender (N)**^**#**^									
Males (264752)	22.80%	19.60%	24.40%	8.20%	8.30%	2.50%	79.30%	82.30%	1.50%
	(22.6–23)	(19.5–19.8)	(24.3–24.6)	(8.1–8.3)	(8.2–8.4)	(2.5–2.6)	(79.2–79.5)	(82.2–82.5)	(1.5–1.6)
Females (326283)	30.80%	23.50%	33.10%	11.90%	11.90%	2.70%	79.60%	82.30%	1.70%
	(30.6–31)	(23.4–23.6)	(32.9–33.2)	(11.8–12)	(11.8–12.1)	(2.6–2.7)	(79.4–79.7)	(82.2–82.4)	(1.7–1.8)
**Database (N)**									
CPRD database only (591037)	27.20%	21.80%	29%	10%	10.10%	2.60%	55.40%		1.6%
	(27.1–27.3)	(21.6–21.9)	(28.9–29.2)	(10.0–10.1)	(10.0–10.1)	(2.6–2.7)	(55.3–55.5)		(1.6–1.7)
CPRD & Linked data (591037)			29.20%	10.30%	10.30%		79.40%	82.30%	
			(29.1–29.3)	(10.2–10.3)	(10.2–10.4)		(79.3–79.5)	(82.2–82.4)	

* row percentages IMD index of multiple deprivation CPRD Clinical Practice Research Datalink

^**#**^ 2 individuals had indeterminate gender

The most incompletely recorded social factor was immigration status which available for only 4,187 (0.7%, data not shown) of the study population when country of birth codes were used alone. However, the additional use of ‘first language’ codes with country of birth codes more than doubled the information, to 1.6% (n = 9,713) of the study population (Table 1).

Religion was the second most poorly recorded factor, available for only 2.6% (n = 15,449) of study individuals (Table 1). Data on place of residence was recorded for 10.3% of the population, whereas living alone (yes/no) and marital status were recorded for nearly a third of the study population (29.2% and 27.2%, respectively).

The contribution of data from linked datasets to completeness of recording was particularly important for ethnicity, which showed a ~45% improvement (increasing from ~55% to ~80%, Table 1) after including linked hospitalisation data, and for IMD data (which was only available as linked data). For other social factors, there was hardly any evidence of improvement in completeness of recording from the linked data compared to using CPRD alone (Table 1).

The utilisation of family number in providing information for individuals who had no data for living alone, cohabitation, care home residence and marital status in either CPRD or HES showed that there was much higher completeness of recoding for living alone (70% versus 29%), cohabitation (60% versus 22%) and marital status (60% versus 27%) when information from family number was included (S3 Table, S4 Table and S5 Table). In contrast, family number contributed little to the completeness of recording of care home residence (11% versus 10%), irrespective of definitions used (S4 Table).

### Representativeness

**Fig 2:**
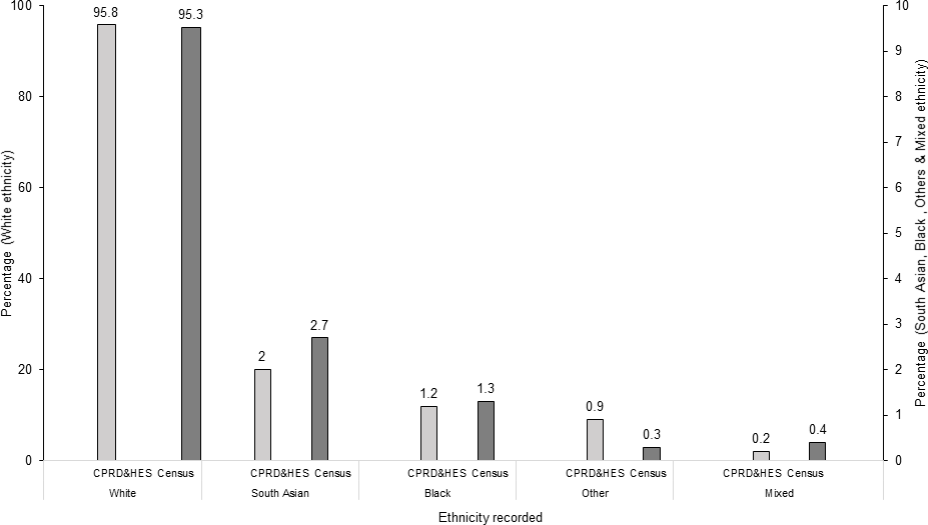
Comparing ethnicity recording (denominator: Those with available data) in electronic health records with English Census 2011. Abbreviations: CPRD Clinical Practice Research Datalink HES Hospital Episode Statistics.

Amongst those with ethnicity data available, (Table 2), White ethnicity was recorded for the majority (~95%) and the ethnic composition of the study population was comparable to the English Census data[34] (Fig 2). In contrast, amongst the small number of individuals with available data on religion, 85% (n = 13,074) were recorded as Christians (Table 2), with an over-representation of the minority religion categories in CPRD (Table 2) compared to Census data[33], for example Muslim (3.1% in CPRD versus 1.3% in the Census), Hindu (2.5% versus 0.8%), Jewish (1.6% versus 0.7%) and Sikh (1.1% versus 0.4%). When Muslim religion was considered as a binary variable, using the entire study population as the denominator and assuming those without a code were non-Muslim, there was appreciable under-recording of Muslim status (n = 481, 0.1%) compared to English Census (1.3%).[33]

**Table 2:** Social factors in Clinical Practice Research Datalink: Recording and categorisation.

Social factors recorded* and their categorisation (N = Total number with information available)		CPRD only N (%)*	CPRD & linked data N (%)*
Marital status (N = 160812)	Single	7291 (4.5%)	No further information from linked data
	Married/Civil	108921 (67.7%)	
	Widow/er	30459 (18.9%)	
	Divorced	7446 (4.6%)	
	Separated	2100 (1.3%)	
	Partner uncategorised/other^#^	4595 (2.9%)	
Living arrangements: Cohabitation (N = 128573)	No	14666 (11.4%)	No further information from linked data
	Yes	113907 (88.6%)	
Living arrangements: living alone CPRD (N = 171625); CPRD & linked data (N = 172590)	No	165914 (96.7%)	166896 (96.7%)
	Yes	5711 (3.3%)	5694 (3.3%)
Residence: place CPRD (N = 59263); CPRD & linked data (N = 60638)	Care home	28318 (47.8%)	28876 (47.6%)
	Sheltered	1001 (1.7%)	1272 (2.1%)
	Household	29371 (49.5%)	29296 (48.3%)
	Others	573 (1%)	1194 (2%)
Residence: homelessness CPRD (N = 59435); CPRD & linked data (N = 60809)	No	59342 (99.8%)	60717 (99.8%)
	Yes	93 (0.2%)	92 (0.2%)
Religion (N = 15449)	Christian	13074 (84.6%)	No further information from linked data
	Buddhist	40 (0.3%)	
	Hindu	389 (2.5%)	
	Jewish	249 (1.6%)	
	Muslim	481 (3.1%)	
	Sikh	169 (1.1%)	
	Other	31 (0.2%)	
	No religion	1016 (6.6%)	
Ethnicity CPRD (N = 327420); CPRD & linked data (N = 469557)	White	311466 (95.1%)	449668 (95.7%)
	South Asian	7688 (2.4%)	9316 (2%)
	Black	4686 (1.4%)	5483 (1.2%)
	Other	2727 (0.8%)	4045 (0.9%)
	Mixed	853 (0.3%)	1045 (0.2%)
Index of Multiple Deprivation (N = 486426)	Least deprived	No information from CPRD	119826 (24.6%)
	2		126957 (26.1%)
	3		101068 (20.8%)
	4		81978 (16.9%)
	Most deprived		56597 (11.6%)
Immigration status (N = 9713)	Not immigrant	1847 (19%)	No further information from linked data
	Immigrant	7866 (81%)	

* Total study population = 591037 CPRD Clinical Practice Research Datalink IMD

^#^ Due to very small numbers in `Partner: other’ category the data are combined with `Partner: uncategorised’

**Fig 3:**
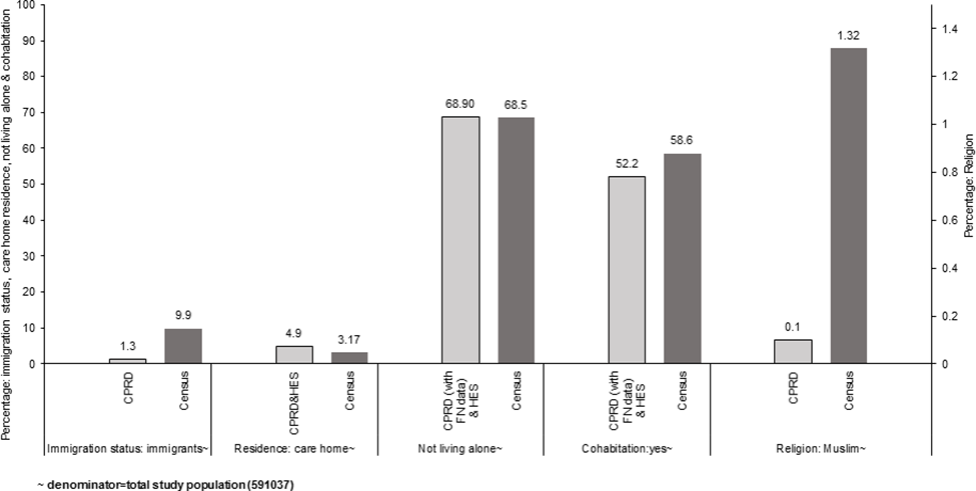
Comparing recording of immigration status, care home residence, not living alone, cohabitation and religion in electronic health records and English Census 2011. Abbreviations CPRD Clinical Practice Research Datalink HES Hospital Episode Statistics FN family number.

Similarly, among those with data on immigrant status, there was marked over-representation of immigrants (n = 7,866, ~81% of the total) among those with recorded data (Table 2), but under-representation when immigrant status was considered as a binary variable (1.3% of the total study population (Fig 3) compared to 9.9% non-UK born individuals in the English Census).[35]

For living arrangements, amongst those with available data, the proportion of individuals recorded as living in a household (~50%, Table 2) was under-reported in CPRD compared to English Census data (in which ~96% of people aged ≥65 years were recorded as living in household) and living in a care home was over-reported (~48%) compared to Census (3.2%).[36, 37] However, once care home residence was categorised as a binary yes/no variable, representativeness improved markedly; in the total study population, 4.9% of individuals were categorised care home residents compared to 3.2% in the English Census data (Fig 3).[37]

The data from EHR for marital status amongst those with non-missing data were also not comparable to the Census data, [38–40] with 68% being recorded as married or in a civil partnership, compared to 55.9% in the Census data. Data were comparable for the sub-categories of: ‘single’ (4.5% versus 5.5% in the Census) and ‘separated’ (1.3% versus 1.2%), but there were small number of individuals in both these sub-categories, making it difficult to draw any conclusions.

The number of individuals with a code indicating that they were homeless was also very small, representing just 0.02% (n = 92) of total study population. There were no corresponding data in the 2011 Census, but data for statutory homelessness and homelessness prevention and relief data (2013) from local authorities in England for individuals aged ≥65 years showed that the proportion of homeless individuals accepted for assistance was 0.01%,[41] providing a minimum estimate of the true proportion of homeless individuals (as not all would have been accepted for assistance).

Amongst those with available data, individuals categorised as those not living alone and as cohabiting were both over-represented in the data (96.7% and 88.6% respectively, Table 2). When considered as a binary variable using the entire study population as denominator, these factors were under-represented (28% and 19% respectively) compared to the Census data (68.5% and 58.6%, respectively).[39, 40] However, when information from family number was added, the percentage of those not living alone (68.9%) or cohabiting (52.2%) were fairly comparable (68.5% and 58.6% respectively) to the Census data (Fig 3).[39, 40]

For deprivation (Table 2), the data showed a slightly lower proportion of study population from the two most deprived quintiles of IMD status, suggesting that older patients in the practices consenting for linkage with deprivation data tended to be from more affluent areas. This is in contrast to a previous study which suggested that overall, including patients of all ages, those in linked CPRD IMD data are comparable to the UK population.[42]

### Timeliness

**Fig 4:**
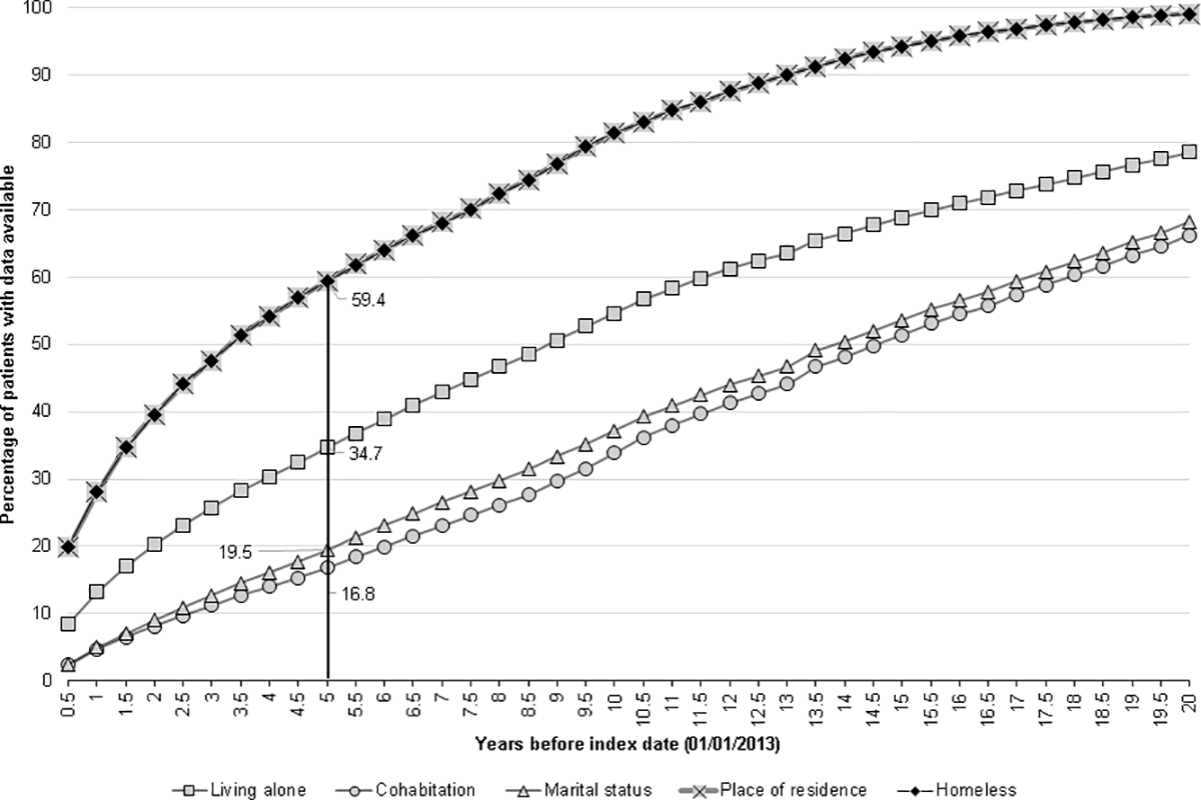
Timeliness of recording of living alone, cohabitation, marital status and place of residence. *data from family number analysis excluded

The recording of time-varying social factors in relation to the index date varied considerably (Fig 4). Amongst those who had information available, 34.7% of individuals had data on whether they lived alone recorded within 5 years of the index date if data from family number was not included, but this decreased to about 20% if family number data were also considered (Fig 4 and S1 Fig). The equivalent percentages for marital status, without and with family number data were 19.5% and 13.5%, respectively. (Fig 4 and S2 Fig). Little difference (58.8% versus 59.5%) was observed for recording of residence within this defined period for analyses including and excluding family number (Fig 4 and S1 Fig).

The equivalent figures for timeliness when the entire study population (n = 591037) was considered, varied from 3.7% for cohabitation status to 14.2% for living alone data (including use of family number, S3 Fig).

## Discussion and conclusions

This study presents the methodology for ascertaining social factors utilising one of the largest collections of primary care EHR in the world. This involved drawing up detailed code lists, utilising multiple files within CPRD and in the linked hospitalisation data to maximise ascertainment, and devising algorithms to time-update variables and to deal with discordant recording. Wide variation in the completeness of recording of social factors was noted, ranging from 1.6% for immigration status to ~82% for deprivation. Overall, the completeness for recording was better amongst females and older individuals, perhaps reflecting a higher consultation rates amongst this demographic group.[43]

The influence of GP incentivisation on completeness of recording of social factors was evident in the recording of ethnicity, an important factor for describing disease burden and for ascertaining health inequities. In 2006 GPs were incentivised to record ethnicity for all newly registered patients[44] and in year 2008 this was extended for all registered patients including the recording of first language spoken.[45] However, this incentivisation was withdrawn on 31 March 2011[25] and we found signs of a downward trend in ethnicity recording from 2011 onwards. The ethnicity data from the present study were available for 79% of the study population and when compared to Census data, were found to be representative of the English population. These results are comparable to an earlier study that reported ethnicity recording in CPRD and linked data for all age groups combined, which found completeness of recoding to be ~78% and ethnicity composition comparable to UK Census.[24]

Immigration status and religion were poorly recorded in these data, and living arrangements were also sub-optimally recorded. Among those with data, a higher than expected proportion were of minority religion, immigrant status or living in a care home, suggesting that GPs are more likely to record these specific social characteristics. When these factors were considered as binary variables (present or absent) in the entire study population, comparison with Census data suggested that care home status may indeed be well recorded. This is perhaps not surprising, as these individuals may be fragile and have higher healthcare needs, necessitating more attendances and interventions. In contrast, being of Muslim religion or an immigrant appeared to be under-recorded. However, our use of “first language” codes may have preferentially captured immigrants from specific countries, whilst under-ascertaining English-speaking individuals born in countries such as the Republic of Ireland, North America, Australasia and the Caribbean, who comprised of ~34% of non-UK born individuals in the 2011 Census.[35] This under-ascertainment may be exacerbated for individuals who moved to the UK many decades previously. Thus, CPRD data may be better for capturing recent arrivals to the UK who are not native English speakers. Homelessness was also under-recorded in these datasets, representing just 0.02% (n = 92) of total study population. Although the proportion of homeless individuals registered with GP has increased (63% in 2002 to 90% in 2014), the poor recording of homelessness status in these data is likely to reflect difficulties encountered by homeless individuals in accessing GP services.[46, 47]

Our findings show that completeness of recording was enhanced by use of multiple sources within datasets, as well as use of linked data. Living in a care home was recorded by GPs in the clinical, referral and test files, consultation data, additional clinical details and could be inferred from the family number, with additional information provided in the hospital data. Similarly, living arrangements such as cohabitation and living alone, the latter an important indicator of morbidity and mortality,[14, 48] were well captured for the study population (~60% and 70%, respectively) when Read code and family number data from CPRD and HES data were combined. Other studies have utilised family number to identify care home residence[26] and cohabitation status.[27, 28, 49] We found that addition of data from family number improved completeness and representativeness of recording of whether a patient lived alone or cohabited, but at the potential expense of timeliness of recording and misclassification. The family number variable is generated by the general practice software when a patient registers with a GP or moves address, assigning the same number to individuals with the same address (Personal communications via email CPRD Knowledge Centre). As the date of updating family number is not captured directly, we took the patient’s registration date as a conservative estimate of when these data were recorded. Patients can move in or out of households and this information may not be captured by the practice, and patients sharing households may be registered at different practices, so that cohabitation status and living alone may be wrongly assigned. For this reason, we used family number to supplement information only when it was unavailable from other sources.

Other social characteristics of patients may have been misclassified in these routinely collected medical records–either due to mis-recording or because patients’ status changed over time and this was not updated. Even factors considered time-invariant in this study may not necessarily have been so; for example, individuals may change their religion. A further point is that the codes used for determining social factors in general practice have not been validated except for ethnicity.[50] We could not examine other social factors that may be associated with uptake of healthcare interventions and health inequities but that were not recorded in these data, such as education, income, housing, social class, social relationships and cultural beliefs.[1, 51]

The significance of determining social factors in assessing the quality of healthcare and value-based payments to healthcare providers have been recognised, for example in a 2017 report published in the United States.[51, 52] A rise in multi-morbidity and frailty amongst older individuals due to population ageing will also increase the need for assessing social factors for delivering equitable healthcare. The CPRD database is used internationally for a wide range of public health studies, and HES includes nation-wide data used extensively for National Health Service (NHS) based research in the UK. Our methods will be thus of interest to researchers using these data. The underlying methods of this study could also be adapted for use in other UK primary care databases. The broader methodological approach utilised in this study such as to investigate the timeliness and the representativeness of these factors in electronic health data by comparing to a national standard such as Census data should be generalizable to other countries with EHR. Our study shows that linked general practice data can be used to ascertain individuals’ ethnicity, deprivation status, care home residence, and whether they live alone. However, other factors such as religion and immigration status are incompletely captured and as mentioned earlier some relevant social characteristics are not recorded in these data. Improvement in completeness and quality of recording of these factors could be achieved by GP incentivisation and use of unambiguous codes. The effect of GP incentivisation was evident in the recording of ethnicity in CPRD which increased from ~30% in the period prior to incentivisation to >80% during the period of incentivisation.[24] A similar approach could be used for other social factors that are currently poorly captured in these data. Increasing health care providers’ awareness about the role of social factors in disease burden and uptake of interventions should also help to improve recording of these factors. Linkages of general practice records with other population based data such as the Census could also greatly enhance the availability of information on social factors.

## Supporting information

S1 Table Sources of information for social factors in linked Clinical Practice Research Datalink. (DOCX)

S2 Table Code lists for social factors. (DOCX)

S3 Table Pattern of completeness for social factors recording (N = 591037(100%)). (DOCX)

S4 Table Time varying social factors and data source: Discordant information on same date and missing event dates (total study population 591037(100%)). (DOCX)

S5 Table Additional information obtained from using family number. (DOCX)

S1 Fig Timeliness of recording of living alone and residence: Comparing data from Clinical Practice Research Datalink (CPRD) and Hospital Episodes Statistics (HES) with data obtained from CPRD, HES and family number. (DOCX)

S2 Fig Timeliness of recording of cohabitation and marital status: Comparing data from Clinical Practice Research Datalink (CPRD) and Hospital Episodes Statistics (HES) with data obtained from CPRD, HES and family number. (DOCX)

S3 Fig Proportion of total study population (n = 591037) with recording of time varying social factors within 5 years of index date (01/01/2013). (DOCX)
