# Supplementary material for: Identifying social factors amongst older individuals in linked electronic health records: An assessment in a population based study
Source: PLoS One. 2017 Nov 30;12(11):e0189038. doi: 10.1371/journal.pone.0189038 (PMC5708811; doi:10.1371/journal.pone.0189038)
Supplement: S1 Table — (DOCX) [file pone.0189038.s001.docx]

S1 Table Sources of information for social factors in linked Clinical Practice Research Datalink

| Social factors | CPRD data files | | | | | | | | HES | Deprivation data |
| --- | --- | --- | --- | --- | --- | --- | --- | --- | --- | --- |
|  | Patient file | Patient file (family number) | Consultation file | Clinical file | Additional clinical details | Immunisation file | Referral file | Test file |  |  |
| Religion | - | - | - | Medocdes | - | Medcodes | Medcodes | Medcodes | - | - |
| Ethnicity | - | - | - | Medcodes | - | Medcodes | Medcodes | Medcodes | Ethnos | - |
| Immigration status | - | - | - | Medcodes | - | Medcodes | Medcodes | Medcodes | - | - |
| IMD | - | - | - | - | - | - | - | - | - | Yes |
| Type of residence including homelessness | - | Yes | Consultation Type:30 & 31 | Medcodes | Entity Type: 132 | Medcodes | Medcodes | Medcodes | Admisorc | - |
| Living alone/ cohabitation | Derived from marital status | Yes | Consultation Type:30 & 31 | Medcodes | Entity Type: 132 | Medcodes | Medcodes | Medcodes | Admisorc | - |
| Marital status | Marital status | Yes | - | Medcodes | Entity Type: 98 | Medcodes | Medcodes | Medcodes | - | - |

CPRD Clinical Practice Research Datalink HES Hospital Episodes Statistics IMD index of multiple deprivation
