# Supplementary material for: Identifying social factors amongst older individuals in linked electronic health records: An assessment in a population based study
Source: PLoS One. 2017 Nov 30;12(11):e0189038. doi: 10.1371/journal.pone.0189038 (PMC5708811; doi:10.1371/journal.pone.0189038)
Supplement: S2 Table — (DOCX) [file pone.0189038.s002.docx]

S2 Table Code lists for social factors

1. Codelist for Clinical Practice Research Datalink
2. Marital status

| **Medcode** | **Readcode** | **Readterm** |
| --- | --- | --- |
| 207 | 13M1.00 | Death of spouse |
| 333 | 13H4200 | Marital conflict |
| 723 | 13H4.12 | Marital stress |
| 838 | 13H3000 | Divorce proceedings |
| 954 | 13H4100 | Marital breakdown |
| 1328 | 6124.00 | Partner had vasectomy |
| 1349 | 13H4.00 | Marital problems |
| 1522 | 1334.00 | Divorced |
| 1540 | 13H4212 | Marital disharmony |
| 1580 | 1332.11 | Remarried |
| 2093 | 13H2.00 | Separation |
| 2159 | 13H3100 | Divorce proceedings pending |
| 3111 | 13HP100 | Girlfriend relationship problem |
| 3321 | SN56300 | Battered wife |
| 3394 | 13W9.00 | Single parent family |
| 3483 | 13HD.00 | Violent spouse |
| 3551 | 13H4211 | Marital discord |
| 3719 | 13L6.00 | Spouse unwell |
| 3988 | 1332.00 | Married |
| 4204 | 1333.00 | Separated |
| 4312 | 1335.00 | Widowed |
| 4531 | 13HP000 | Boyfriend relationship problem |
| 4565 | 6741.00 | Marital counselling |
| 4925 | 1333.13 | Wife left home |
| 5055 | 1333.12 | Husband left home |
| 6056 | 13H3.00 | Divorce |
| 6104 | 13HP.00 | Relationship problems |
| 7419 | 1332.12 | Newly wed |
| 7869 | 13H1.00 | Marriage |
| 8470 | 13IL100 | Wife pregnant |
| 9112 | 13HX.00 | New relationship |
| 9551 | ZV61100 | [V]Marital problems |
| 9612 | 1311.00 | Housewife |
| 9910 | 13H3.11 | Divorce problems |
| 10330 | 13L3.11 | Alcoholic spouse |
| 11103 | 1331.11 | Single - unmarried |
| 11251 | 13MG.00 | Death of wife |
| 12076 | ZU14111 | Husband died |
| 12325 | ZU14100 | Death of husband |
| 13001 | 6123.00 | No partner at present |
| 15020 | 13HG.11 | Spouse left home |
| 15115 | 13HH.15 | Looks after chronically sick spouse |
| 15313 | 13H4300 | Maladjustment to married life |
| 15404 | 13L3.13 | Husband alcoholic |
| 15527 | 133C.00 | Widower |
| 15777 | 13HV311 | Spouse committed infidelity |
| 15824 | 13L1.11 | Disabled spouse |
| 15950 | 13H5.12 | Spouse returned home |
| 16262 | 13H6.11 | Unmarried parent |
| 16315 | 133G.00 | Common-law husband |
| 16344 | 8C81.12 | Artificial insemin by husband |
| 16552 | 13HH.13 | Looks after chronically sick husband |
| 17538 | 13IL300 | Wife alive |
| 17802 | 13MH.00 | Husband died |
| 20079 | 13H6.00 | Single parent |
| 20149 | 13HH.16 | Looks after chronically sick wife |
| 20217 | 13HM.11 | Legal problem with separation |
| 20313 | 13HM.12 | Legal problem with divorce |
| 20536 | 13HV100 | Affair ended |
| 21346 | 8C92.00 | Spouse reassured |
| 21433 | 13HV313 | Husband committed adultery |
| 21860 | 13IL.00 | Health of spouse |
| 21925 | 13HV400 | Seven year itch - marital |
| 22336 | 13L6.11 | Has infirm partner |
| 22909 | 13JK.13 | Partnership problems |
| 22934 | 6124.11 | Partner sterilised |
| 23385 | 13FD100 | Spouse cannot care for patient |
| 23409 | 13HV000 | Affair started |
| 23445 | 13HV200 | Affair unsatisfactory |
| 23508 | SN56400 | Battered husband |
| 23514 | 13HT114 | Wife unable to cope |
| 23858 | ZV61011 | [V]Divorce |
| 23974 | 13IL200 | Wife well |
| 24055 | 13HG.00 | Broken with partner |
| 24769 | 13I7100 | Husband in prison |
| 25097 | 13MI.00 | Death of husband |
| 25149 | 13W9011 | Single parent family - mother |
| 25452 | 13D2.00 | Homeless single person |
| 25503 | 1336.00 | Cohabiting |
| 27385 | 1333.11 | Separated from cohabitee |
| 27434 | 13HV011 | Lover taken |
| 27572 | 13HE.00 | Engaged |
| 28440 | 13MF.00 | Death of partner |
| 28484 | U3N0.00 | [X]Other maltreatment syndromes, by spouse or partner |
| 29543 | 13H5.00 | Marital reconciliation |
| 29544 | 13H4.11 | Marital trouble |
| 30597 | ZV26500 | [V]Artificial insemination from husband |
| 30950 | 13H4213 | Row with wife |
| 31495 | 8H7I.00 | Refer to partner |
| 31678 | 13EF.00 | Divorced couple sharing house |
| 32451 | 67M..00 | Informing partner |
| 32984 | 1F81.00 | Spouse cooks food |
| 33000 | 13Q..12 | Widows pensions |
| 33001 | 131..11 | Occupation of husband |
| 33153 | 1312.00 | House husband |
| 33188 | 13ID.00 | Partner unemployed |
| 34771 | 13HV.00 | Extra-marital problems |
| 36077 | 131..00 | Occupation of spouse |
| 36333 | ZLB4.00 | Seen by marriage guidance counsellor |
| 36947 | 13L7.00 | Partner dying |
| 37113 | U3M0.00 | [X]Neglect and abandonment, by spouse or partner |
| 37265 | 1331.00 | Single |
| 37551 | 13HVZ00 | Extra-marital problems NOS |
| 38325 | 13H5.11 | Cohabitee returned |
| 39292 | 131..12 | Occupation of wife |
| 39474 | 9NA8.00 | Cohabitee made appointment |
| 39651 | 13HV300 | Spouse committed adultery |
| 39879 | 13HV314 | Oil rig wives syndrome |
| 40493 | 13HF.00 | Broken engagement |
| 40866 | 13Q..00 | Widows benefits |
| 41203 | 13L1200 | Spouse is handicapped |
| 42321 | 13H4311 | Spouse unsympathetic |
| 42386 | 133H.00 | Common-law wife |
| 42390 | 13I7300 | Boyfriend arrested |
| 42398 | 13I7200 | Spouse arrested |
| 42400 | 13HV312 | Wife committed adultery |
| 42402 | 13WE.00 | Spouse works away from home |
| 42428 | 1333.14 | Cohabitee left home |
| 45005 | 1AZ5.00 | Fertility problems in partner |
| 45010 | 13Q3.00 | Widows pension |
| 47411 | 13I2.00 | Partner stops work |
| 49666 | 1AZ4.00 | Low sperm count in partner |
| 50149 | 13W7000 | Crime against spouse |
| 50485 | 13HY.00 | First relationship |
| 54096 | 131Z.00 | Occupation of spouse NOS |
| 54816 | 13H4312 | Spouse inattentive |
| 56178 | 13HV012 | Mistress taken |
| 59817 | 9d31.00 | Husband |
| 59829 | 9d32.00 | Wife |
| 60723 | 918j.00 | Partner is informal carer |
| 60821 | 13QZ.00 | Widows benefits NOS |
| 61291 | 1A85.00 | Breast lump detected by partner |
| 61509 | 13Q..11 | Widows allowances |
| 63118 | 1276.11 | Spouse haemophiliac |
| 68095 | 13I7400 | Girlfriend arrested |
| 88373 | 133S.00 | Married/civil partner |
| 91652 | 7E0A300 | Intrauterine insemination superovulation partner sperm |
| 94044 | 133V.00 | Widowed/surviving civil partner |
| 94917 | 13Ir.00 | Partner pregnant |
| 95101 | 9d30.00 | Spouse |
| 96856 | 133T.00 | Divorced/person whose civil partnership has been dissolved |
| 97076 | 6127.00 | Partner had tubal ligation |
| 98130 | 9d33.00 | Cohabitee |
| 98610 | 13Q5.00 | War widows pension |
| 98818 | 13Q1.00 | Widows allowance |
| 99328 | 13I1.00 | Partner begins work |
| 100785 | 68b9.00 | Anten screen, partner tested and no genetic risk identified |
| 101900 | 13I4.00 | Partner works after retirement |
| 102413 | 133b.00 | Partner in relationship |
| 104879 | 133e.00 | Common law partnership |
| 104936 | U3P0.00 | [X]Maltreatment, by spouse or partner |
| 109323 | 13I3.00 | Partner retires |

1. Cohabitation

| **Medcode** | **Readcode** | **Readterm** |
| --- | --- | --- |
| 333 | 13H4200 | Marital conflict |
| 723 | 13H4.12 | Marital stress |
| 954 | 13H4100 | Marital breakdown |
| 1349 | 13H4.00 | Marital problems |
| 1540 | 13H4212 | Marital disharmony |
| 1580 | 1332.11 | Remarried |
| 3321 | SN56300 | Battered wife |
| 3394 | 13W9.00 | Single parent family |
| 3483 | 13HD.00 | Violent spouse |
| 3551 | 13H4211 | Marital discord |
| 3719 | 13L6.00 | Spouse unwell |
| 3988 | 1332.00 | Married |
| 4565 | 6741.00 | Marital counselling |
| 4925 | 1333.13 | Wife left home |
| 5055 | 1333.12 | Husband left home |
| 7419 | 1332.12 | Newly wed |
| 7869 | 13H1.00 | Marriage |
| 8470 | 13IL100 | Wife pregnant |
| 9551 | ZV61100 | [V]Marital problems |
| 9612 | 1311.00 | Housewife |
| 10330 | 13L3.11 | Alcoholic spouse |
| 11103 | 1331.11 | Single - unmarried |
| 13001 | 6123.00 | No partner at present |
| 15115 | 13HH.15 | Looks after chronically sick spouse |
| 15313 | 13H4300 | Maladjustment to married life |
| 15404 | 13L3.13 | Husband alcoholic |
| 15777 | 13HV311 | Spouse committed infidelity |
| 15824 | 13L1.11 | Disabled spouse |
| 15950 | 13H5.12 | Spouse returned home |
| 16262 | 13H6.11 | Unmarried parent |
| 16315 | 133G.00 | Common-law husband |
| 16344 | 8C81.12 | Artificial insemin by husband |
| 16552 | 13HH.13 | Looks after chronically sick husband |
| 17538 | 13IL300 | Wife alive |
| 20079 | 13H6.00 | Single parent |
| 20149 | 13HH.16 | Looks after chronically sick wife |
| 21346 | 8C92.00 | Spouse reassured |
| 21433 | 13HV313 | Husband committed adultery |
| 21860 | 13IL.00 | Health of spouse |
| 21925 | 13HV400 | Seven year itch - marital |
| 23385 | 13FD100 | Spouse cannot care for patient |
| 23508 | SN56400 | Battered husband |
| 23514 | 13HT114 | Wife unable to cope |
| 23974 | 13IL200 | Wife well |
| 24769 | 13I7100 | Husband in prison |
| 25149 | 13W9011 | Single parent family - mother |
| 25452 | 13D2.00 | Homeless single person |
| 25503 | 1336.00 | Cohabiting |
| 27385 | 1333.11 | Separated from cohabitee |
| 29543 | 13H5.00 | Marital reconciliation |
| 29544 | 13H4.11 | Marital trouble |
| 30597 | ZV26500 | [V]Artificial insemination from husband |
| 30950 | 13H4213 | Row with wife |
| 31678 | 13EF.00 | Divorced couple sharing house |
| 32984 | 1F81.00 | Spouse cooks food |
| 33001 | 131..11 | Occupation of husband |
| 33153 | 1312.00 | House husband |
| 34771 | 13HV.00 | Extra-marital problems |
| 36077 | 131..00 | Occupation of spouse |
| 36333 | ZLB4.00 | Seen by marriage guidance counsellor |
| 37113 | U3M0.00 | [X]Neglect and abandonment, by spouse or partner |
| 37265 | 1331.00 | Single |
| 37551 | 13HVZ00 | Extra-marital problems NOS |
| 38325 | 13H5.11 | Cohabitee returned |
| 39292 | 131..12 | Occupation of wife |
| 39474 | 9NA8.00 | Cohabitee made appointment |
| 39651 | 13HV300 | Spouse committed adultery |
| 39879 | 13HV314 | Oil rig wives syndrome |
| 41203 | 13L1200 | Spouse is handicapped |
| 42321 | 13H4311 | Spouse unsympathetic |
| 42386 | 133H.00 | Common-law wife |
| 42398 | 13I7200 | Spouse arrested |
| 42400 | 13HV312 | Wife committed adultery |
| 42402 | 13WE.00 | Spouse works away from home |
| 42428 | 1333.14 | Cohabitee left home |
| 50149 | 13W7000 | Crime against spouse |
| 54096 | 131Z.00 | Occupation of spouse NOS |
| 54816 | 13H4312 | Spouse inattentive |
| 59817 | 9d31.00 | Husband |
| 59829 | 9d32.00 | Wife |
| 60723 | 918j.00 | Partner is informal carer |
| 63118 | 1276.11 | Spouse haemophiliac |
| 88373 | 133S.00 | Married/civil partner |
| 95101 | 9d30.00 | Spouse |
| 98130 | 9d33.00 | Cohabitee |
| 104879 | 133e.00 | Common law partnership |

1. Living arrangement: living alone

| **Medcode** | **Readcode** | **Readterm** |
| --- | --- | --- |
| 464 | 13HT115 | Domestic problems |
| 1123 | 13HQ.00 | In prison |
| 1650 | 13HT100 | Stress at home |
| 2562 | 13D..11 | Homeless |
| 2955 | 1B1K.12 | Lives alone |
| 11504 | ZU33200 | Lives with daughter |
| 12798 | ZU33600 | Lives with father |
| 12807 | ZU33100 | Lives with children |
| 13355 | 13F1.00 | Independant housing, not alone |
| 13356 | 13F2.00 | Lives alone - help available |
| 13357 | 13F3.00 | Lives alone -no help available |
| 13358 | 13FH.00 | Lives with relatives |
| 15416 | 13F7300 | Lives in a childrens home |
| 15691 | 13F3100 | Lives alone needs housekeeper |
| 15700 | 13JS.00 | Works away from home |
| 15840 | 13F7100 | Lives in a welfare home |
| 17279 | 13FH000 | Elderly relative lives with family |
| 19610 | 13FJ.00 | Independent housing, lives alone |
| 20155 | 13HT113 | Home unsettled |
| 21405 | 13F7400 | Admitted to a children's home |
| 22249 | ZU3..11 | Lives with |
| 22336 | 13L6.11 | Has infirm partner |
| 22503 | ZU33300 | Lives with son |
| 23575 | 13FD.00 | No carers, though not alone |
| 25167 | ZU31.00 | Lives alone |
| 25452 | 13D2.00 | Homeless single person |
| 25715 | 8He1.00 | Referral to intermediate care - community rehabilitation |
| 26177 | 8He0.00 | Referral to intermediate care - hospital at home |
| 28448 | ZU33500 | Lives with mother |
| 30200 | ZV60611 | [V]Boarding school resident |
| 30965 | 13E6.00 | Overcrowded in house |
| 31385 | 13F8100 | Long stay hospital inpatient |
| 31678 | 13EF.00 | Divorced couple sharing house |
| 32753 | 13D3.11 | Tramp |
| 32774 | 13D1.00 | Homeless family |
| 32882 | 8He..00 | Referral to intermediate care |
| 33006 | 1311.11 | Homemaker |
| 33994 | ZW63200 | Staying with carer |
| 34506 | 13FL.00 | Living rough |
| 36418 | ZV60300 | [V]Person living alone |
| 36730 | Z37C.00 | Provision of special residential school |
| 36809 | ZU33.00 | Lives with family |
| 36947 | 13L7.00 | Partner dying |
| 36968 | ZU33400 | Lives with parents |
| 40822 | ZU33700 | Lives with grandparents |
| 41388 | 13D5.00 | Vagrant |
| 42533 | ZU26100 | Number of dependants in household |
| 42654 | ZU35.00 | Lives with companion |
| 43393 | ZU33800 | Lives with grandfather |
| 43911 | ZU33900 | Lives with grandmother |
| 47577 | ZW63100 | Living with carer |
| 47591 | 13FS.00 | Long stay hospital inpatient |
| 49138 | ZV63212 | [V]Delayed discharge - nursing home vacancy awaited |
| 50111 | 13HH.11 | Cares for mentally handicapped dependent |
| 50206 | 8O0A.00 | Provision of special residential school |
| 50994 | 13HH.18 | Looks after physically handicapped dependent |
| 51193 | ZU32.00 | Lives with friends |
| 52682 | 6992.00 | Prison medical examination |
| 53343 | ZU3..12 | LW - Lives with |
| 55276 | ZU37.00 | Lives in a community |
| 57438 | ZU32100 | Lives with friend |
| 59330 | T776.00 | Place of occurrence of accident or poisoning, prison |
| 59523 | 13D3.12 | Vagabond |
| 61385 | 9b1C.00 | Hospice - independent |
| 66549 | 13EA.00 | Multiple occupancy |
| 67112 | 9k6..00 | Homeless - enhanced services administration |
| 67187 | 13FM.00 | Sleeping in night shelter |
| 68005 | 13FV.00 | Lives in a welfare home |
| 70021 | ZU36.00 | Lives as companion |
| 71339 | 0A82.00 | Companion |
| 71663 | ZU37200 | Lives in boarding school |
| 73177 | ZLG6100 | Discharge to long stay hospital |
| 86390 | 13FY.00 | Lives in a children's unit |
| 90547 | ZU34.00 | Lives with lodger |
| 94886 | 13Il.00 | Subject to interim supervision order under Children Act 1989 |
| 95555 | ZU37300 | Lives in a commune |
| 95880 | 13It.00 | Lives with grandmother |
| 96605 | 9k60.00 | Homeless - enhanced service completed |
| 97138 | 13Is.00 | Lives with grandfather |
| 99907 | ZV60011 | [V]Hobo |
| 101400 | 13Zr.00 | Lives with immunocompromised person |
| 101582 | 9b0t.00 | Children's home visit note |
| 103510 | ZV60014 | [V]Tramp |
| 103553 | ZU37100 | Lives in a school community |
| 104962 | 13D8.00 | Length of time homeless |
| 106972 | 13IZ000 | Lives with adoptive parents |
| 107393 | 9Ngr.00 | Under care of homeless advocacy service |
| 107733 | 13IZ200 | Lives with biological parents |
| 107809 | 918F200 | Lives with carer |
| 109673 | 13IZ100 | Lives with biological parent and step parent |

1. Residence

| **Medcode** | **Readcode** | **Readterm** |
| --- | --- | --- |
| 1123 | 13HQ.00 | In prison |
| 2562 | 13D..11 | Homeless |
| 6855 | 9491.00 | Patient died at home |
| 6859 | 9N1F.00 | Seen in warden sup home |
| 6991 | 9493.00 | Patient died in nursing home |
| 7101 | 9N1F.12 | Seen in old people's home |
| 7653 | 9N1G.00 | Seen in nursing home |
| 10120 | 9N1C.00 | Seen in own home |
| 10993 | ZLG4.00 | Discharge to nursing home |
| 11419 | 13F7200 | Lives in an old peoples home |
| 11504 | ZU33200 | Lives with daughter |
| 11949 | 13F4.00 | Warden attended |
| 12798 | ZU33600 | Lives with father |
| 12807 | ZU33100 | Lives with children |
| 13355 | 13F1.00 | Independant housing, not alone |
| 13357 | 13F3.00 | Lives alone -no help available |
| 13358 | 13FH.00 | Lives with relatives |
| 13359 | 13F6100 | Lives in a nursing home |
| 13360 | 13F6.00 | Nursing/other home |
| 13361 | 13F4.11 | Lives in warden controlled accommodation |
| 13562 | ZV70317 | [V]Old age home admission medical |
| 15691 | 13F3100 | Lives alone needs housekeeper |
| 15700 | 13JS.00 | Works away from home |
| 15840 | 13F7100 | Lives in a welfare home |
| 17279 | 13FH000 | Elderly relative lives with family |
| 18291 | 13EC.00 | House in poor repair |
| 19610 | 13FJ.00 | Independent housing, lives alone |
| 21280 | 13F5200 | Resident in part III accomodation |
| 22503 | ZU33300 | Lives with son |
| 24494 | ZV60600 | [V]Institution resident |
| 24756 | 13KD.00 | Owner-occupier |
| 24815 | 13K8.00 | House rented from council |
| 24816 | Z177C00 | Residential care |
| 24828 | Z177F00 | Nursing home care |
| 24910 | 13KA.00 | House rented-private landlord |
| 24956 | 13FK.00 | Lives in a residential home |
| 25143 | 13K6.00 | Houseowner - no mortgage |
| 25452 | 13D2.00 | Homeless single person |
| 26177 | 8He0.00 | Referral to intermediate care - hospital at home |
| 26720 | 13FB.00 | Living in lodgings |
| 26812 | 9494.00 | Patient died in resid.inst.NOS |
| 27360 | 13F5100 | Part III accomodation arranged |
| 27425 | 13F5.00 | Part III accommodation |
| 27936 | 8HE6.00 | Delayed discharge to nursing home |
| 27968 | 13F7.00 | Residential institution |
| 28448 | ZU33500 | Lives with mother |
| 28773 | ZV60700 | [V]Sheltered housing |
| 30200 | ZV60611 | [V]Boarding school resident |
| 30807 | 13F4000 | Resident in sheltered accommodation |
| 31385 | 13F8100 | Long stay hospital inpatient |
| 31678 | 13EF.00 | Divorced couple sharing house |
| 31951 | 13F9.00 | Living in hostel |
| 32448 | 13EH100 | Harrassment by landlord |
| 32753 | 13D3.11 | Tramp |
| 32774 | 13D1.00 | Homeless family |
| 33006 | 1311.11 | Homemaker |
| 33153 | 1312.00 | House husband |
| 33994 | ZW63200 | Staying with carer |
| 34506 | 13FL.00 | Living rough |
| 34794 | 13F9.11 | Living in sheltered accomodatn |
| 35040 | ZLG5.00 | Discharge to sheltered housing |
| 35172 | 9N1E.00 | Seen in warden sup flat |
| 35187 | 9N1D.00 | Seen in warden sup house |
| 35279 | 9N1H.00 | Seen in Elderly Mentaly Infirm home |
| 35716 | 13FA.00 | Living in B&B accommodation |
| 36096 | 13F5.11 | Part 3 accomodation |
| 36730 | Z37C.00 | Provision of special residential school |
| 36809 | ZU33.00 | Lives with family |
| 36905 | ZLG5100 | Discharge to warden controlled accommodation |
| 36968 | ZU33400 | Lives with parents |
| 37829 | U195100 | [X]Victim of volcanic eruption occurrn in resident instit'n |
| 39311 | 9492.00 | Patient died in part 3 accom. |
| 39685 | 13K7.00 | Houseowner with mortgage |
| 40822 | ZU33700 | Lives with grandparents |
| 41188 | 13FC.11 | Lives in a bedsit |
| 41388 | 13D5.00 | Vagrant |
| 41986 | 699Z.00 | Exam. for institution NOS |
| 42191 | ZLG3.00 | Discharge to residential home |
| 42533 | ZU26100 | Number of dependants in household |
| 42654 | ZU35.00 | Lives with companion |
| 43057 | 13FG.00 | Squatter |
| 43393 | ZU33800 | Lives with grandfather |
| 43709 | ZV70H00 | [V]Examination for admission to residential institutions |
| 43911 | ZU33900 | Lives with grandmother |
| 43915 | ZLG4100 | Discharge to private nursing home |
| 44053 | 699..00 | Examination for institution |
| 45650 | T704.00 | Place of occurrence of accident/poisoning, residential house |
| 46222 | T774.00 | Place of occurrence of accident/poisoning, old people's home |
| 46303 | U10z100 | [X]Unspecified fall, occurrence in residential institution |
| 46588 | 13K9.00 | House rented from housing ass. |
| 46642 | 9b79.00 | Other residential care homes managed by local authority |
| 47577 | ZW63100 | Living with carer |
| 47591 | 13FS.00 | Long stay hospital inpatient |
| 47609 | T77..00 | Place of accident or poisoning, residential institution |
| 47685 | ZV6y200 | [V]Other boarder in health-care facility |
| 48549 | ZLG3100 | Discharge to private residential home |
| 48733 | U198100 | [X]Victim of flood, occurrence in residential institution |
| 48805 | U120100 | [X]Hit struck kick twist bit/scratch anoth pers resid instit |
| 48932 | U125100 | [X]Bitten/struck by oth mammal occurrn in resident instit'n |
| 49138 | ZV63212 | [V]Delayed discharge - nursing home vacancy awaited |
| 49210 | U101100 | [X]Fall same level from slip trip + stumb occ resid instit |
| 49681 | 13FX.00 | Lives in care home |
| 50206 | 8O0A.00 | Provision of special residential school |
| 50792 | 9N1F.11 | Seen in Part 3 accomodation |
| 51193 | ZU32.00 | Lives with friends |
| 51495 | 13FC.00 | Living in bedsitter |
| 51851 | U104100 | [X]Fall whle carried/supported oth persons occ resid instit |
| 52249 | 13FQ.00 | Lives on council site |
| 52466 | U10A100 | [X]Fall on + from stair + step occurrnce resident instit'n |
| 52682 | 6992.00 | Prison medical examination |
| 52881 | U291.00 | [X]Intent self harm by sharp object occ resident instit'n |
| 53140 | Z177D00 | Local authority residential care |
| 53600 | U12A100 | [X]Contct wth plant thorn+spine+sharp leave occ resid instit |
| 54260 | U3F1.00 | [X]Assault by blunt object occurrn in resident institution |
| 54735 | 13EC.11 | Slum housing |
| 54948 | ZLG5200 | Discharge to part III accommodation |
| 55276 | ZU37.00 | Lives in a community |
| 56326 | U3K1.00 | [X]Assault by bodily force occurrn in residential institut'n |
| 56969 | T77z.00 | Accident/poisoning occurred in residential institution NOS |
| 57438 | ZU32100 | Lives with friend |
| 59330 | T776.00 | Place of occurrence of accident or poisoning, prison |
| 59523 | 13D3.12 | Vagabond |
| 59548 | 13FT.00 | Lives in an old peoples home |
| 59653 | 6991.00 | Geriatric home admission exam. |
| 60404 | U221.00 | [X]Intent self harm by drowning/submersn occ resid instit'n |
| 60684 | U2A1.00 | [X]Intent self harm by blunt object occ resident instit'n |
| 61385 | 9b1C.00 | Hospice - independent |
| 62522 | U3z1.00 | [X]Assault by unspecified means occurrn resident institut'n |
| 64410 | U211.00 | [X]Intent self harm by hangng strangult/suffoct resid instit |
| 65445 | 13FP.00 | Lives on private site |
| 66122 | 13F5111 | Part 3 accomodation arranged |
| 66599 | U152100 | [X]Exposure to unspecif electric current occ resid instit'n |
| 66656 | U128100 | [X]Bitten/struck by crocodil/alligatr occ in resid instit'n |
| 66922 | U108100 | [X]Fall involv other furniture occurrn resident institut'n |
| 67112 | 9k6..00 | Homeless - enhanced services administration |
| 67187 | 13FM.00 | Sleeping in night shelter |
| 67586 | U241.00 | [X]Int self harm rifl s'gun/lrg frarm disch occ resid instit |
| 67903 | U105100 | [X]Fall involvng wheelchair occurrence residential instit'n |
| 67930 | 13FG.11 | Illegal tennant |
| 68005 | 13FV.00 | Lives in a welfare home |
| 69028 | ZLG3200 | Discharge to part III residential home |
| 69762 | U106100 | [X]Fall involving bed occurrence in residential institution |
| 70021 | ZU36.00 | Lives as companion |
| 70848 | 13FW.00 | Living in temporary housing |
| 71663 | ZU37200 | Lives in boarding school |
| 72474 | U10J100 | [X]Other fall on same level, occurrnce in resident instit'n |
| 72716 | U143100 | [X]Inhalation of gastric contents occurrn resident instit'n |
| 72838 | 13FR.00 | Lives on unofficial site |
| 73083 | 9b0Y.00 | Nursing home visit note |
| 73101 | U3L1.00 | [X]Sexual assault by bodily force occurrn resident instit'n |
| 73177 | ZLG6100 | Discharge to long stay hospital |
| 73321 | 9b1P.00 | Nursing home |
| 87882 | U2y1.00 | [X]Intent self harm by oth specif means occ resid instit'n |
| 90547 | ZU34.00 | Lives with lodger |
| 91941 | U11Q100 | [X]Foreign body enter into/thr eye/natrl orif, resid instit |
| 92265 | U197100 | [X]Victim of cataclysmic storm occurrn in resident instit'n |
| 92315 | U3y1.00 | [X]Assault by oth specif means occurrn resident institution |
| 93837 | U2C1.00 | [X]Int self harm jump/lying befr mov obje occ resid instit'n |
| 93865 | U11H100 | [X]Explosn+ruptur of pressr tyre pipe/hose occ resid instit |
| 93998 | 9b0i.00 | Residential home visit note |
| 94070 | 8O24.00 | Provision of continuing care in nursing home |
| 95555 | ZU37300 | Lives in a commune |
| 95661 | U116100 | [X]Contact wth knife sword/dagger occurrn in resid instit'n |
| 95880 | 13It.00 | Lives with grandmother |
| 96605 | 9k60.00 | Homeless - enhanced service completed |
| 96663 | U112100 | [X]Strikng against/struck by other object occ in resid inst |
| 97138 | 13Is.00 | Lives with grandfather |
| 97757 | 13D7.00 | Sofa surfer - person of no fixed abode |
| 99091 | U122100 | [X]Crush push/step on by crowd/humn stampede occ resid inst |
| 99110 | U10F100 | [X]Fall from cliff, occurrence in residential institution |
| 99120 | U193100 | [X]Victim of lightning, occurrn in residential institution |
| 99148 | 9b7A.00 | Other residential care home man voluntary/private agents |
| 99453 | U156100 | [X]Expos unspecif type of radiatn occurrn resident instit'n |
| 99598 | U114100 | [X]Contact with lifting+transmissn dev NEC occ resid instit |
| 99907 | ZV60011 | [V]Hobo |
| 100246 | ZVu5700 | [X]Other boarder in health care facility |
| 100389 | U321.00 | [X]Assault by pesticides occurrn in residential institution |
| 100710 | U10D100 | [X]Fall from out of/thro buildng/struct occ resid instit'n |
| 101003 | 9NFR.00 | Home visit request by residential institution |
| 101078 | 949D.00 | Patient died in care home |
| 101400 | 13Zr.00 | Lives with immunocompromised person |
| 102230 | M270100 | Nursing home acquired pressure ulcer |
| 102493 | 8Ht..00 | Admission to nursing home |
| 103138 | U3E1.00 | [X]Assault by sharp object occurrn in resident institution |
| 103285 | 9b70.00 | Client's or patient's home |
| 103461 | 133c.00 | Hospital at home patient |
| 103510 | ZV60014 | [V]Tramp |
| 103553 | ZU37100 | Lives in a school community |
| 104962 | 13D8.00 | Length of time homeless |
| 105063 | U127100 | [X]Bittn/stung by nven insct+oth nven arthrop occ resid inst |
| 106027 | 13KD.11 | Lives in own home |
| 106285 | U126100 | [X]Contact wth marine animal occurrn in resident institut'n |
| 106972 | 13IZ000 | Lives with adoptive parents |
| 107072 | U144100 | [X]Inhal+ingest food caus obst resp tract occ resid instit'n |
| 107393 | 9Ngr.00 | Under care of homeless advocacy service |
| 107733 | 13IZ200 | Lives with biological parents |
| 107757 | 9NFW.00 | Care home visit |
| 107809 | 918F200 | Lives with carer |
| 107927 | U1B4100 | [X]Lack of water, occurrence in residential institution |
| 108702 | 13D6.00 | Lives in squat |
| 109437 | TD17200 | Accident due to fall from burning convalescent home |
| 109673 | 13IZ100 | Lives with biological parent and step parent |

v) Religion

| **Medcode** | **Readcode** | **Readterm** |
| --- | --- | --- |
| 2053 | 1357 | Jehovah's Witness |
| 12477 | 1355 | Jewish |
| 12622 | 135Y.00 | Spiritualist |
| 12685 | 1358 | Hindu |
| 19559 | 135D.00 | Religion, none |
| 24263 | 1351 | Church of England |
| 24268 | 1352 | Roman Catholic |
| 24273 | 135S.00 | Buddhist |
| 24297 | 135B.00 | Sikh |
| 24341 | 1359 | Islam |
| 24669 | 135A.00 | Christian |
| 25594 | 135F.00 | Baptist |
| 25996 | 1359.11 | Muslim |
| 29954 | 1356 | Christian Scientist |
| 31044 | 135J.00 | Church of Scotland |
| 31585 | 135N.00 | Plymouth Brethren |
| 39701 | 135M.00 | Society of Friends |
| 39751 | 135G.00 | Methodist |
| 39839 | 1354 | Atheist |
| 46063 | 9iF6.00 | Jewish - ethnic category 2001 Census |
| 47091 | 9iF7.00 | Muslim - ethnic category 2001 Census |
| 47329 | 135W.00 | Salvation Army |
| 47959 | 135K.00 | Pentecostal |
| 47961 | 135L.00 | Evangelical |
| 47962 | 135C.00 | Mixed religion |
| 47968 | 135P.00 | Agnostic |
| 47971 | 1351.11 | Anglican |
| 47972 | 135I.00 | Presbyterian |
| 47998 | 135d.00 | Orthodox Christian |
| 47999 | 135V.00 | Jainism |
| 48000 | 135H.00 | United Reform Church |
| 49658 | 9iF8.00 | Sikh - ethnic category 2001 Census |
| 50229 | 135T.00 | Rastafarian |
| 52201 | 135O.00 | Christadelphian |
| 56127 | 9iF5.00 | Hindu - ethnic category 2001 Census |
| 57757 | 1353 | Nonconformist |
| 58665 | 1359100 | Sunni muslim |
| 63872 | 9iF4.00 | Buddhist - ethnic category 2001 Census |
| 64041 | 1359000 | Shiite muslim |
| 64056 | 135b.00 | Pagan |
| 64057 | 135c.00 | Mormon |
| 64058 | 135a.00 | Moravian religion |
| 92227 | 135X.00 | Eastern Catholic |
| 99738 | 13zA.00 | Protestant |
| 100125 | 13zp.00 | Church of England, follower of religion |
| 100433 | 13z2.00 | Armenian Orthodox |
| 100435 | 13yH.00 | Follower of Goddess tradition |
| 100497 | 13yX.00 | Mennonite |
| 100522 | 13zJ.00 | Church of Scotland, follower of religion |
| 100526 | 13yc.00 | Seventh Day Adventist |
| 100536 | 13z6.00 | Scottish Episcopalian |
| 100713 | 13yQ.00 | Pure Land Buddhist |
| 100794 | 13zC.00 | French Protestant |
| 100867 | 135l.00 | African religion, follower of religion |
| 100951 | 135m.00 | Yoruba, follower of religion |
| 101115 | 13zG.00 | Lutheran |
| 101118 | 13yB.00 | Ancestral worship |
| 101144 | 135z.00 | New age practitioner |
| 101384 | 13zi.00 | Orthodox Jew |
| 101650 | 135i.00 | Baha'i |
| 101828 | 13z5.00 | Ukrainian Catholic |
| 101837 | 13yP.00 | Zen Buddhist |
| 101856 | 13z3.00 | Greek Orthodox |
| 101905 | 13y8.00 | Black magic |
| 102123 | 135x.00 | Native American religion, follower of religion |
| 102124 | 13z9.00 | Catholic: non Roman Catholic |
| 102238 | 13yi.00 | Judaic Christian |
| 102253 | 135v.00 | Radha Soami |
| 102459 | 13zE.00 | Follower of United Reformed Church |
| 102498 | 13yD.00 | Wiccan |
| 102646 | 135e.00 | Shinto |
| 102697 | 13yy.00 | Romanian Orthodox |
| 102902 | 13zF.00 | Quaker |
| 102956 | 13za.00 | Sanatana Dharma |
| 102991 | 13zd.00 | Shakti Hindu |
| 103060 | 135j.00 | Druze |
| 103225 | 135t.00 | Satanist |
| 103376 | 13y5.00 | Chondogyo |
| 103692 | 13ys.00 | Russian Orthodox |
| 103742 | 13yH.11 | Goddess |
| 103845 | 13y3.00 | Humanist |
| 103929 | 13zD.00 | Free Church of Scotland |
| 104111 | 13z1.00 | Bulgarian Orthodox |
| 104329 | 135v.11 | Sant Mat |
| 104723 | 13zH.00 | Congregationalist |
| 104737 | 13zl.00 | Ashkenazi Jew |
| 104893 | 13yl.00 | Christian Existentialist |
| 104965 | 13ze.00 | Smarta Hindu |
| 105120 | 135w.00 | Pantheist |
| 105407 | 13y4.00 | Deist |
| 105517 | 13yF.00 | Occultist |
| 105548 | 13y1.00 | Kabbalist |
| 105874 | 135q.00 | Taoist |
| 106119 | 135p.00 | Unitarian Universalist |
| 106797 | 13yt.00 | Ethiopian Orthodox Tewahedo |
| 107174 | 135Z.11 | Rastafarian |
| 107288 | 13yh.00 | Messianic Jew |
| 107636 | 13zP.00 | Reformed Christian |
| 107810 | 13yv.00 | Ukrainian Orthodox |
| 107871 | 13yG.00 | Heathen |
| 107962 | 13zh.00 | Reform Jew |
| 108011 | 135k.00 | Ahmadi |
| 108014 | 13ym.00 | Celtic Christian |
| 108015 | 13yj.00 | Christian Spiritualist |
| 108066 | 13yZ.00 | Free Church |
| 108225 | 13yC.00 | Zoroastrian |
| 108497 | 13zg.00 | Arya Samaj Hindu |
| 108571 | 13yn.00 | Celtic Orthodox Christian |
| 109080 | 13yN.00 | Mahayana Buddhist |
| 109590 | 13z8.00 | Church in Wales |

vi) Country of birth

| **Medcode** | **Readcode** | **Readterm** |
| --- | --- | --- |
| 4114 | 13ZC.00 | Immigrant |
| 8929 | ZV70314 | [V]Immigration medical |
| 9144 | 13Z6800 | Speaks English poorly |
| 9292 | 133L.00 | Immigrant |
| 9627 | 13ZN.00 | Asylum seeker |
| 11552 | 13e..00 | Country of birth (Asian) |
| 12458 | 13gf.00 | Born in South Africa |
| 12713 | 13eG.00 | Born in Iraq |
| 22294 | 13lZ.00 | Main spoken language Turkish |
| 23523 | 13Z6000 | English as a second language |
| 24295 | 13Z6500 | Language Punjabi |
| 24296 | 13Z6300 | Language Hindi |
| 24403 | 13ZB.00 | Refugee |
| 24691 | 13Z6600 | Language Urdu |
| 24712 | 13Z6200 | Language Gujurati |
| 24741 | 13Z6100 | Language Bengali |
| 24881 | 13lC.00 | Main spoken language Polish |
| 25007 | 13eH.00 | Born in Israel |
| 25008 | 13go.00 | Born in Zimbabwe |
| 25092 | 13eY.00 | Born in Philippines |
| 25133 | 13gi.00 | Born in Tanzania |
| 25256 | 13dl.00 | Born in Yugoslavia |
| 25410 | 13b0.00 | Vietnamese language |
| 25423 | 13lS.00 | Main spoken language Albanian |
| 25472 | 13l2.00 | Main spoken language Cantonese |
| 25609 | 13lx.00 | Main spoken language Thai |
| 25616 | 13lp.00 | Main spoken language Malayalam |
| 25632 | ZV70516 | [V]Refugee health examination |
| 25664 | 13gC.00 | Born in Congo |
| 25665 | 13l5.00 | Main spoken language French |
| 25730 | 13dM.00 | Born in Kosovo |
| 25752 | 13dC.00 | Born in England |
| 25802 | 13l1.00 | Main spoken language Bengali |
| 25829 | 13lE.00 | Main spoken language Punjabi |
| 25995 | 13eW.00 | Born in Pakistan |
| 26078 | 13lP.00 | Main spoken language Shona |
| 26196 | 13Z6400 | Language Pashtu |
| 26247 | 13lH.00 | Main spoken language Spanish |
| 26334 | 13eo.00 | Born in Vietnam |
| 26335 | 13lb.00 | Main spoken language Vietnamese |
| 26337 | 13l0.00 | Main spoken language Arabic |
| 26361 | 13lL.00 | Main spoken language Urdu |
| 26426 | 13eF.00 | Born in Iran |
| 26463 | 13dA.00 | Born in Czech Republic |
| 26464 | 13l3.00 | Main spoken language Czech |
| 28301 | 13dP.00 | Born in Lithuania |
| 28529 | 13eg.00 | Born in Syria |
| 30224 | 13gY.00 | Born in Niger |
| 30606 | 13f5.00 | Born in Canada |
| 30800 | 13d..00 | Country of birth (European) |
| 32053 | 13f..00 | Country of birth (American) |
| 32055 | 13d0.00 | Born in Albania |
| 32058 | 13e8.00 | Born in China |
| 32060 | 13e0.00 | Born in Afghanistan |
| 32061 | 13h0.00 | Born in Australia |
| 32062 | 13jC.00 | Born in Trinidad and Tobago |
| 32065 | 13db.00 | Born in Scotland |
| 32067 | 13gJ.00 | Born in Ghana |
| 32068 | 13gl.00 | Born in Uganda |
| 32070 | 13eI.00 | Born in Japan |
| 32072 | 13fL.00 | Born in USA |
| 32074 | 13h1.00 | Born in New Zealand |
| 32075 | 13f3.00 | Born in Brazil |
| 32076 | 13j6.00 | Born in Jamaica |
| 32079 | 13g0.00 | Born in Algeria |
| 32080 | 13dH.00 | Born in Greece |
| 32081 | 13dF.00 | Born in France |
| 32082 | 13eD.00 | Born in India |
| 32085 | 13dG.00 | Born in Germany |
| 32089 | 13j2.00 | Born in Barbados |
| 32090 | 13fN.00 | Born in Venezuela |
| 32094 | 13dW.00 | Born in Poland |
| 32097 | 13de.00 | Born in Spain |
| 32098 | 13df.00 | Born in Sweden |
| 32099 | 13gM.00 | Born in Ivory Coast |
| 32102 | 13g..00 | Country of birth (African) |
| 32103 | 13di.00 | Born in Ukraine |
| 32105 | 13dk.00 | Born in Wales |
| 32108 | 13gV.00 | Born in Morocco |
| 32111 | 13gZ.00 | Born in Nigeria |
| 32112 | 13gN.00 | Born in Kenya |
| 32113 | 13dK.00 | Born in Ireland |
| 32114 | 13ek.00 | Born in Turkey |
| 32115 | 13gS.00 | Born in Malawi |
| 32116 | 13dL.00 | Born in Italy |
| 32117 | 13dU.00 | Born in Northern Ireland |
| 32119 | 13dc.00 | Born in Slovakia |
| 32120 | 13fB.00 | Born in Grenada |
| 32125 | 13e3.00 | Born in Bangladesh |
| 32127 | 13eb.00 | Born in Russia |
| 32128 | 13eM.00 | Born in Kyrgyzstan |
| 32131 | 13j9.00 | Born in St. Lucia |
| 32135 | 13ej.00 | Born in Thailand |
| 32139 | 13dD.00 | Born in Estonia |
| 32140 | 13gU.00 | Born in Mauritius |
| 32144 | 13ec.00 | Born in Saudi Arabia |
| 32150 | 13dh.00 | Born in The Netherlands |
| 32157 | 13ed.00 | Born in Singapore |
| 32158 | 13gG.00 | Born in Ethiopia |
| 32160 | 13dX.00 | Born in Portugal |
| 32162 | 13gW.00 | Born in Mozambique |
| 32166 | 13fJ.00 | Born in Peru |
| 32167 | 13g5.00 | Born in Burundi |
| 32168 | 13gn.00 | Born in Zambia |
| 32169 | 13d7.00 | Born in Bulgaria |
| 32171 | 13eP.00 | Born in Malaysia |
| 32173 | 13dE.00 | Born in Finland |
| 32186 | 13dB.00 | Born in Denmark |
| 32189 | 13ge.00 | Born in Somalia |
| 32190 | 13d9.00 | Born in Cyprus |
| 32197 | 13gd.00 | Born in Sierra Leone |
| 32201 | 13fF.00 | Born in Mexico |
| 32202 | 13e7.00 | Born in Chechnya |
| 32207 | 13gI.00 | Born in Gambia |
| 32217 | 13eT.00 | Born in Nepal |
| 32220 | 13eC.00 | Born in Hong Kong |
| 32233 | 13gX.00 | Born in Namibia |
| 32237 | 13gP.00 | Born in Liberia |
| 32242 | 13dN.00 | Born in Latvia |
| 32245 | 13ef.00 | Born in Sri Lanka |
| 32254 | 13e6.00 | Born in Burma |
| 32255 | 13g7.00 | Born in Cameroon |
| 32260 | 13g1.00 | Born in Angola |
| 32273 | 13e2.00 | Born in Bahrain |
| 32293 | 13k4.00 | Born in Seychelles |
| 32301 | 13j0.00 | Born in Antigua and Barbuda |
| 32303 | 13f7.00 | Born in Columbia |
| 32304 | 13jB.00 | Born in Togo |
| 32309 | 13gc.00 | Born in Senegal |
| 32311 | 13f9.00 | Born in Ecuador |
| 32313 | 13d2.00 | Born in Austria |
| 32325 | 13f0.00 | Born in Argentina |
| 32331 | 13ga.00 | Born in Rwanda |
| 32333 | 13gE.00 | Born in Egypt |
| 32342 | 13f4.00 | Born in British Guyana |
| 32345 | 13dZ.00 | Born in Romania |
| 32347 | 13gL.00 | Born in Guinea Republic |
| 32352 | 13d4.00 | Born in Belgium |
| 32361 | 13e1.00 | Born in Armenia |
| 32369 | 13j4.00 | Born in Dominican Republic |
| 32390 | 13eh.00 | Born in Taiwan |
| 32397 | 13d6.00 | Born in Bosnia - Herzegovnia |
| 32417 | 13k..00 | Country of birth (Pacific) |
| 32427 | 13lF.00 | Main spoken language Russian |
| 32456 | 13lG.00 | Main spoken language Somali |
| 32688 | 13dI.00 | Born in Hungary |
| 32728 | 13lD.00 | Main spoken language Portuguese |
| 32741 | 13gk.00 | Born in Tunisia |
| 32776 | 13lB.00 | Main spoken language Mandarin |
| 32807 | 13dg.00 | Born in Switzerland |
| 36794 | 13gg.00 | Born in Sudan |
| 36852 | 13lW.00 | Main spoken language Japanese |
| 36862 | 13lt.00 | Main spoken language Serbian |
| 36980 | 13lQ.00 | Main spoken language Italian |
| 37197 | 13gR.00 | Born in Madagascar |
| 38075 | 13fD.00 | Born in Guyana |
| 38117 | 13gA.00 | Born in Chad |
| 39974 | 13dS.00 | Born in Moldavia |
| 41209 | 13gm.00 | Born in Zaire |
| 41210 | 13d8.00 | Born in Croatia |
| 41211 | 13eO.00 | Born in Lebanon |
| 41213 | 13dV.00 | Born in Norway |
| 41217 | 13eL.00 | Born in Kuwait |
| 41228 | 13ee.00 | Born in South Korea |
| 41230 | 13f2.00 | Born in Bolivia |
| 41233 | 13d5.00 | Born in Belorussia |
| 41280 | 13fE.00 | Born in Honduras |
| 41289 | 13eE.00 | Born in Indonesia |
| 41290 | 13j3.00 | Born in Cuba |
| 41291 | 13f6.00 | Born in Chile |
| 41292 | 13eK.00 | Born in Kazakhstan |
| 41297 | 13eX.00 | Born in Palestine |
| 41302 | 13el.00 | Born in Turkmenistan |
| 41304 | 13dd.00 | Born in Slovenia |
| 41311 | 13eJ.00 | Born in Jordan |
| 41312 | 13dJ.00 | Born in Iceland |
| 41316 | 13g3.00 | Born in Botswana |
| 41318 | 13g2.00 | Born in Benin |
| 41327 | 13h..00 | Country of birth (Australasian) |
| 41337 | 13d3.00 | Born in Azerbaijan |
| 41341 | 13gQ.00 | Born in Libya |
| 41344 | 13ep.00 | Born in Yemen |
| 41350 | 13gh.00 | Born in Swaziland |
| 41351 | 13eS.00 | Born in Mongolia |
| 41354 | 13e9.00 | Born in Democratic People's Republic of Korea |
| 41356 | 13ea.00 | Born in Republic of Korea |
| 41357 | 13dY.00 | Born in Republic of Ireland |
| 41364 | 13dR.00 | Born in Malta |
| 41365 | 13gK.00 | Born in Guinea Bissau |
| 41367 | 13fM.00 | Born in Uruguay |
| 41372 | 13em.00 | Born in United Arab Emirates |
| 41399 | 13fI.00 | Born in Paraguay |
| 41402 | 13en.00 | Born in Uzbekistan |
| 42635 | 13k0.00 | Born in Fiji |
| 42639 | 13eZ.00 | Born in Qatar |
| 46014 | 13lN.00 | Main spoken language Kurdish |
| 46029 | 13l8.00 | Main spoken language Hindi |
| 46325 | 13lY.00 | Main spoken language Lithuanian |
| 46861 | 13lK.00 | Main spoken language Tamil |
| 46973 | 13ln.00 | Main spoken language Lingala |
| 46974 | 13lV.00 | Main spoken language Greek |
| 47007 | 13li.00 | Main spoken language French Creole |
| 47029 | 13lO.00 | Main spoken language Farsi |
| 47073 | 133Q.00 | Family reunion immigrant |
| 47399 | 13n2.00 | Reads Punjabi |
| 47400 | 13n9.00 | Reads Cantonese |
| 47402 | 13n0.00 | Reads Arabic |
| 47404 | 13n7.00 | Reads Urdu |
| 47559 | 13k6.00 | Born in Tonga |
| 47627 | 13lJ.00 | Main spoken language Sylheti |
| 47628 | 13ld.00 | Main spoken language Amharic |
| 47630 | 13lR.00 | Main spoken language German |
| 47631 | 13lw.00 | Main spoken language Tagalog |
| 47641 | 13lI.00 | Main spoken language Swahili |
| 47643 | 13lu.00 | Main spoken language Sinhala |
| 47644 | 13ly.00 | Main spoken language Tigrinya |
| 47646 | 13lm.00 | Main spoken language Igbo |
| 48002 | 13l6.00 | Main spoken language Gujerati |
| 48029 | 6951.00 | Immigration examination |
| 48297 | 13k5.00 | Born in Solomon Islands |
| 49402 | 13eB.00 | Born in Georgia |
| 49907 | 13j..00 | Country of birth (Atlantic) |
| 51778 | 13eV.00 | Born in Oman |
| 52200 | 13b4.00 | Mirpuri language |
| 52204 | 13nD.00 | Reads Hindi |
| 52209 | 13n8.00 | Reads Bengali |
| 54409 | 13lM.00 | Main spoken language Yoruba |
| 54410 | 13lT.00 | Main spoken language Croatian |
| 54413 | 13lc.00 | Main spoken language Akan |
| 54414 | 13lf.00 | Main spoken language Dutch |
| 54415 | 13lX.00 | Main spoken language Korean |
| 54416 | 13l9.00 | Main spoken language Iba |
| 54417 | 13lv.00 | Main spoken language Swedish |
| 56879 | 13lh.00 | Main spoken language Flemish |
| 57186 | 13eA.00 | Born in East Timor |
| 57189 | 13k7.00 | Born in Tuvalu |
| 57341 | 13nE.00 | Reads Chinese |
| 57343 | 13n5.00 | Reads Spanish |
| 57345 | 13n1.00 | Reads Portuguese |
| 57462 | 13b3.00 | Creole language |
| 57755 | 13ll.00 | Main spoken language Hebrew |
| 57758 | 13lq.00 | Main spoken language Norwegian |
| 58192 | 13j5.00 | Born in Haiti |
| 58193 | 13l7.00 | Main spoken language Hausa |
| 58525 | 13nC.00 | Reads French |
| 58527 | 13g4.00 | Born in Burkina Faso |
| 58528 | 13n3.00 | Reads Russian |
| 58531 | 13n4.00 | Reads Somali |
| 58533 | 13j1.00 | Born in Bahamas |
| 58537 | 13nF.00 | Reads Polish |
| 58552 | 13nA.00 | Reads Czech |
| 58643 | 13lr.00 | Main spoken language Pashto |
| 59657 | 13gH.00 | Born in Gabon |
| 62298 | 13e5.00 | Born in Brunei |
| 63923 | 13fH.00 | Born in Panama |
| 63927 | 13gj.00 | Born in The Gambia |
| 63932 | 13sA.00 | English as a second language |
| 63943 | 13fA.00 | Born in El Salvador |
| 64120 | 13k3.00 | Born in Papua New Guinea |
| 64391 | 13lj.00 | Main spoken language Gaelic |
| 64948 | 13ls.00 | Main spoken language Patois |
| 64949 | 13gp.00 | Born in Eritrea |
| 64984 | 13f8.00 | Born in Costa Rica |
| 65310 | 13eR.00 | Born in Mali |
| 65503 | 69D8.00 | Exam. of refugee |
| 66551 | 13jA.00 | Born in St. Vincent |
| 66553 | 13j8.00 | Born in St. Kitts and Nevis |
| 66560 | 13eU.00 | Born in North Korea |
| 66564 | 13nW.00 | Reads Greek |
| 66565 | 13nR.00 | Reads Italian |
| 66685 | 13la.00 | Main spoken language Ukrainian |
| 66826 | 13n6.00 | Reads Tamil |
| 68778 | 13nS.00 | Reads German |
| 68866 | 13gD.00 | Born in Djibouti |
| 69131 | 13dQ.00 | Born in Luxembourg |
| 69135 | 13j7.00 | Born in Puerto Rico |
| 69139 | 13lg.00 | Main spoken language Ethiopian |
| 69143 | 13gT.00 | Born in Mauritania |
| 69153 | 13lo.00 | Main spoken language Luganda |
| 69426 | 13fK.00 | Born in Suriname |
| 69431 | 13eN.00 | Born in Laos |
| 69560 | 13g8.00 | Born in Cape Verde Islands |
| 69806 | 13fC.00 | Born in Guatemala |
| 71190 | 13dT.00 | Born in Monaco |
| 72379 | 13lA.00 | Main spoken language Kutchi |
| 74892 | 13fG.00 | Born in Nicaragua |
| 90860 | 13nH.00 | Reads Farsi |
| 90868 | 13nG.00 | Reads Lithuanian |
| 91328 | 13eQ.00 | Born in Maldives |
| 91419 | 13nY.00 | Reads Turkish |
| 91420 | 13nQ.00 | Reads Kurdish |
| 91422 | 13lk.00 | Main spoken language Hakka |
| 91423 | 13nJ.00 | Reads Chinese - Traditional |
| 93443 | 13nc.00 | Reads Pashto |
| 93444 | 13nf.00 | Reads Tigrinya |
| 93462 | 13nK.00 | Reads Gujarati |
| 93569 | 13nX.00 | Reads Japanese |
| 93697 | 13gO.00 | Born in Lesotho |
| 93893 | 13nh.00 | Reads Burmese |
| 93923 | 13gF.00 | Born in Equatorial Guinea |
| 93935 | 13d1.00 | Born in Andorra |
| 94050 | 13f1.00 | Born in Belize |
| 94072 | 13ng.00 | Reads Bulgarian |
| 94906 | 13Zd.00 | Failed asylum seeker |
| 95590 | 13nM.00 | Reads Chinese - Simplified |
| 95593 | 13nV.00 | Reads Croatian |
| 95708 | 13dO.00 | Born in Liechtenstein |
| 95775 | 13nZ.00 | Reads Vietnamese |
| 95897 | 13u0.00 | Main spoken language Bulgarian |
| 95940 | 13ur.00 | Main spoken language Latvian |
| 95968 | 13lT.11 | Main spoken language Serbo-Croatian |
| 95969 | 13lt.11 | Main spoken language Serbo-Croatian |
| 95970 | 13lO.11 | Main spoken language Persian |
| 95974 | 13lu.11 | Main spoken language Sinhalese |
| 95978 | 13w1.00 | Main spoken language Nepali |
| 95985 | 13nm.00 | Reads Malay |
| 96041 | 13ua.00 | Main spoken language Hungarian |
| 96146 | 13uh.00 | Main spoken language Irish |
| 96147 | 13lE.11 | Main spoken language Panjabi |
| 96148 | 13u5.00 | Main spoken language Afrikaans |
| 96152 | 13u1.00 | Main spoken language Romanian |
| 96163 | 13wL.00 | Main spoken language Telugu |
| 96223 | 13wR.00 | Main spoken language Twi |
| 96230 | 13wG.00 | Main spoken language Slovenian |
| 96240 | 13wD.00 | Main spoken language Sindhi |
| 96267 | 13ux.00 | Main spoken language Marathi |
| 96268 | 13uj.00 | Main spoken language Kannada |
| 96289 | 13uN.00 | Main spoken language Danish |
| 96290 | 13u6.00 | Main spoken language Armenian |
| 96295 | 13t..00 | Born in British overseas territory |
| 96296 | 13na.00 | Reads Amharic |
| 96317 | 13w5.00 | Main spoken language Quechua |
| 96370 | 13uv.00 | Main spoken language Maltese |
| 96376 | 13uu.00 | Main spoken language Malay |
| 96485 | 13le.00 | Main spoken language Brawa |
| 96558 | 13wM.00 | Main spoken language Tibetan |
| 96559 | 13uG.00 | Main spoken language Burmese |
| 96560 | 13uT.00 | Main spoken language Finnish |
| 96611 | 13us.00 | Main spoken language Macedonian |
| 96634 | 13wN.00 | Main spoken language Tongan |
| 96636 | 13g6.00 | Born in Cambodia |
| 96784 | 13wT.00 | Main spoken language Uzbek |
| 96805 | 13nj.00 | Reads Indonesian |
| 96824 | 13dm.00 | Born in former Yugoslav Republic of Macedonia |
| 96857 | 13u2.00 | Main spoken language Oromo |
| 96858 | 13w2.00 | Main spoken language Occitan |
| 96868 | 13u4.00 | Main spoken language Afar |
| 96873 | 13wa.00 | Main spoken language Zulu |
| 96877 | 13w..00 | Supplemental main language spoken |
| 96928 | 13uQ.00 | Main spoken language Estonian |
| 97015 | 13uk.00 | Main spoken language Kashmiri |
| 97038 | 13uz.00 | Main spoken language Mongolian |
| 97039 | 13wW.00 | Main spoken language Wolof |
| 97041 | 13wQ.00 | Main spoken language Turkmen |
| 97083 | 13u9.00 | Main spoken language Azerbaijani |
| 97131 | 13wH.00 | Main spoken language Sundanese |
| 97136 | 13no.00 | Reads Ndebele |
| 97212 | 13uX.00 | Main spoken language Georgian |
| 97273 | 13wX.00 | Main spoken language Xhosa |
| 97274 | 13uc.00 | Main spoken language Indonesian |
| 97297 | 13w3.00 | Main spoken language Oriya |
| 97298 | 9NUC.11 | Persian language interpreter needed |
| 97390 | 13e4.00 | Born in Bhutan |
| 97439 | 13wB.00 | Main spoken language Southern Sotho |
| 97440 | 13w6.00 | Main spoken language Romansh |
| 97574 | 13uB.00 | Main spoken language Basque |
| 97595 | 13ul.00 | Main spoken language Kazakh |
| 97612 | 13v..00 | Born French overseas region department collectivity territor |
| 97644 | 9NUz.00 | Bulgarian language interpreter needed |
| 97685 | 13wP.00 | Main spoken language Tsonga |
| 97789 | 13uP.00 | Main spoken language Esperanto |
| 97997 | 13uK.00 | Main spoken language Catalan |
| 98038 | 13t1.00 | Born in Bermuda |
| 98062 | 9Nmm.00 | Burmese language interpreter needed |
| 98070 | 13uZ.00 | Main spoken language Guarani |
| 98132 | 13up.00 | Main spoken language Lao |
| 98194 | 13um.00 | Main spoken language Kinyarwanda |
| 98215 | 13uy.00 | Main spoken language Moldavian |
| 98228 | 13nN.00 | Reads Swahili |
| 98229 | 13nn.00 | Reads Mongolian |
| 98255 | 13uw.00 | Main spoken language Maori |
| 98285 | 13w4.00 | Main spoken language Filipino |
| 98369 | 13nP.00 | Reads Yoruba |
| 98510 | 13uY.00 | Main spoken language Kalaallisut |
| 98530 | 13da.00 | Born in San Marino |
| 98604 | 13ub.00 | Main spoken language Icelandic |
| 98762 | 13u8.00 | Main spoken language Aymara |
| 98809 | 9NmQ.00 | Hungarian language interpreter needed |
| 98841 | 9NUy.00 | Romanian language interpreter needed |
| 98942 | 13nb.00 | Reads Lingala |
| 99119 | 13dj.00 | Born in Vatican City |
| 99258 | 13g9.00 | Born in Central African Republic |
| 99431 | 13jD.00 | Born in Dominica |
| 99712 | 13wS.00 | Main spoken language Uighur |
| 99740 | 13nd.00 | Reads Serbian |
| 99794 | 9Nn1.00 | Tsonga language interpreter needed |
| 100007 | 13ei.00 | Born in Tajikistan |
| 100010 | 13nT.00 | Reads Albanian |
| 100011 | 13ui.00 | Main spoken language Javanese |
| 100013 | 13uL.00 | Main spoken language Slovak |
| 100438 | 9NmA.00 | Macedonian language interpreter needed |
| 100517 | 13dn.00 | Born in Serbia |
| 100707 | 13uR.00 | Main spoken language Faeroese |
| 100714 | 13uS.00 | Main spoken language Fijian |
| 100716 | 13ug.00 | Main spoken language Inuktitut |
| 100743 | 13uW.00 | Main spoken language Galician |
| 100759 | 9Nn7.00 | Slovenian language interpreter needed |
| 100813 | 9NmM.00 | Interlingue language interpreter needed |
| 100828 | 13uH.00 | Main spoken language Belarusian |
| 100949 | 13uD.00 | Main spoken language Bihari |
| 101038 | 13wb.00 | Main spoken language Konkani |
| 101158 | 13gB.00 | Born in Comoros Islands |
| 101189 | 13uJ.00 | Main spoken language Central Khmer |
| 101220 | 13wA.00 | Main spoken language Dari |
| 101284 | 9NnK.00 | Nepali language interpreter needed |
| 101591 | 13gb.00 | Born in Sao Tome and Principe |
| 101614 | 9Nn4.00 | Telugu language interpreter needed |
| 101620 | 13ut.00 | Main spoken language Malagasy |
| 101659 | 13ud.00 | Main spoken language Interlingua |
| 101761 | 13l9.11 | Main spoken language Iban |
| 101788 | 13uM.00 | Main spoken language Corsican |
| 101814 | 9NUc.11 | Punjabi language interpreter needed |
| 102007 | 13uY.11 | Main spoken language Greenlandic |
| 102127 | 13uC.00 | Main spoken language Dzongkha |
| 102128 | 13uF.00 | Main spoken language Breton |
| 102129 | 13ue.00 | Main spoken language Interlingue |
| 102184 | 13u3.00 | Main spoken language Abkhazian |
| 102218 | 13u7.00 | Main spoken language Assamese |
| 102259 | 13uA.00 | Main spoken language Bashkir |
| 102877 | 13wC.00 | Main spoken language Tswana |
| 103200 | 13uV.00 | Main spoken language Frisian |
| 103219 | 13w7.00 | Main spoken language Samoan |
| 103364 | 13v0.00 | Born in Martinique |
| 103965 | 13k9.00 | Born in Western Samoa |
| 104071 | 13uq.00 | Main spoken language Bamun |
| 104123 | 13Zw.00 | Has United Kingdom student visa |
| 104284 | 13ni.00 | Reads Chechen |
| 104635 | 9NmC.00 | Latvian language interpreter needed |
| 104678 | 9Nmx.00 | Oromo language interpreter needed |
| 104886 | 13wE.00 | Main spoken language Ndebele |
| 104901 | 9Nm6.00 | Brawa language interpreter needed |
| 104983 | 13t2.00 | Born in Anguilla |
| 105079 | 9Nmd.00 | Catalan language interpreter needed |
| 105153 | 133A000 | International student |
| 105523 | 13wV.00 | Main spoken language Tetum |
| 105529 | 13wc.00 | Main spoken language Aragonese |
| 105608 | 13nk.00 | Reads Kinyarwanda |
| 105923 | 13t0.00 | Born in Montserrat |
| 105960 | 13wJ.00 | Main spoken language Tajik |
| 107687 | 133A011 | Overseas student |
| 108184 | 9Nn6.00 | Turkmen language interpreter needed |
| 108271 | 13jE.00 | Born in Aruba |
| 108936 | 13v7.00 | Born in Guadeloupe |
| 109092 | 13ds.00 | Born in Jersey |
| 109093 | 13dr.00 | Born in Guernsey |
| 109226 | 13eq.00 | Born in Christmas Island |
| 109260 | 13dq.00 | Born in Republic of Moldova |
| 109276 | 13t5.00 | Born in St Helena, Ascension and Tristan da Cunha |
| 109457 | 13do.00 | Born in Montenegro |
| 109458 | 13jG.00 | Born in Saint Vincent and the Grenadines |
| 109489 | 9NmE.00 | Kinyarwanda language interpreter needed |
| 109727 | 13t3.00 | Born in British Virgin Islands |
| 109791 | 13kB.00 | Born in American Samoa |
| 109898 | 13dp.00 | Born in Belarus |
| 109992 | 13gq.00 | Born in Democratic Republic of Congo |

vii) Ethnicity

| Medcode | Readcode | Readterm |
| --- | --- | --- |
| 10196 | 9S...00 | Ethnic groups (1991 census) |
| 22467 | 9S1..00 | White |
| 12446 | 9S10.00 | White British |
| 24837 | 9S11.00 | White Irish |
| 12444 | 9S12.00 | Other white ethnic group |
| 26467 | 9S13.00 | White Scottish |
| 26310 | 9S14.00 | Other white British ethnic group |
| 12632 | 9S2..00 | Black Caribbean |
| 12778 | 9S3..00 | Black African |
| 24339 | 9S4..00 | Black, other, non-mixed origin |
| 12452 | 9S41.00 | Black British |
| 57435 | 9S42.00 | Black Caribbean/W.I./Guyana |
| 47950 | 9S42.11 | Black Caribbean |
| 47997 | 9S42.12 | Black West Indian |
| 32100 | 9S42.13 | Black Guyana |
| 41329 | 9S43.00 | Black N African/Arab/Iranian |
| 46812 | 9S43.11 | Black North African |
| 57752 | 9S43.12 | Black Arab |
| 50286 | 9S43.13 | Black Iranian |
| 35412 | 9S44.00 | Black - other African country |
| 47965 | 9S45.00 | Black E Afric Asia/Indo-Caribb |
| 57753 | 9S45.11 | Black East African Asian |
| 57763 | 9S45.12 | Black Indo-Caribbean |
| 48005 | 9S46.00 | Black Indian sub-continent |
| 35350 | 9S47.00 | Black - other Asian |
| 26312 | 9S48.00 | Black Black - other |
| 25676 | 9S5..00 | Black - other, mixed |
| 25623 | 9S51.00 | Other Black - Black/White orig |
| 32165 | 9S52.00 | Other Black - Black/Asian orig |
| 12482 | 9S6..00 | Indian |
| 24690 | 9S7..00 | Pakistani |
| 24740 | 9S8..00 | Bangladeshi |
| 24272 | 9S9..00 | Chinese |
| 30280 | 9SA..00 | Other ethnic non-mixed (NMO) |
| 32110 | 9SA1.00 | Brit. ethnic minor. spec.(NMO) |
| 57764 | 9SA2.00 | Brit. ethnic minor. unsp (NMO) |
| 54593 | 9SA3.00 | Caribbean I./W.I./Guyana (NMO) |
| 57094 | 9SA3.11 | Caribbean Island (NMO) |
| 57075 | 9SA3.12 | West Indian (NMO) |
| 93144 | 9SA3.13 | Guyana (NMO) |
| 24962 | 9SA4.00 | N African Arab/Iranian (NMO) |
| 47285 | 9SA4.11 | North African Arab (NMO) |
| 25082 | 9SA4.12 | Iranian (NMO) |
| 47969 | 9SA5.00 | Other African countries (NMO) |
| 38097 | 9SA6.00 | E Afric Asian/Indo-Carib (NMO) |
| 46818 | 9SA6.11 | East African Asian (NMO) |
| 99316 | 9SA6.12 | Indo-Caribbean (NMO) |
| 39696 | 9SA7.00 | Indian sub-continent (NMO) |
| 26379 | 9SA8.00 | Other Asian (NMO) |
| 24270 | 9SA9.00 | Irish (NMO) |
| 45947 | 9SAA.00 | Greek/Greek Cypriot (NMO) |
| 45955 | 9SAA.11 | Greek (NMO) |
| 47949 | 9SAA.12 | Greek Cypriot (NMO) |
| 32066 | 9SAB.00 | Turkish/Turkish Cypriot (NMO) |
| 32126 | 9SAB.11 | Turkish (NMO) |
| 32069 | 9SAB.12 | Turkish Cypriot (NMO) |
| 12633 | 9SAC.00 | Other European (NMO) |
| 41214 | 9SAD.00 | Other ethnic NEC (NMO) |
| 12696 | 9SB..00 | Other ethnic, mixed origin |
| 47401 | 9SB1.00 | Other ethnic, Black/White orig |
| 32401 | 9SB2.00 | Other ethnic, Asian/White orig |
| 35459 | 9SB3.00 | Other ethnic, mixed white orig |
| 32420 | 9SB4.00 | Other ethnic, other mixed orig |
| 32425 | 9SB5.00 | Black Caribbean and White |
| 32443 | 9SB6.00 | Black African and White |
| 25411 | 9SC..00 | Vietnamese |
| 12429 | 9SD..00 | Ethnic group not given - patient refused |
| 24340 | 9SE..00 | Ethnic group not recorded |
| 32136 | 9SG..00 | Other black ethnic group |
| 12668 | 9SH..00 | Other Asian ethnic group |
| 47601 | 9SI..00 | Irish traveller |
| 12757 | 9SJ..00 | Other ethnic group |
| 45199 | 9SZ..00 | Ethnic groups (census) NOS |
| 23955 | 9T...00 | Ethnicity and other related nationality data |
| 45008 | 9T1..00 | New Zealand ethnic groups |
| 57286 | 9T11.00 | New Zealand European |
| 85509 | 9T11.11 | Pakeha |
| 85505 | 9T12.00 | Other European in New Zealand |
| 32479 | 9T13.00 | New Zealand Maori |
| 64610 | 9T14.00 | Samoan |
| 89910 | 9T15.00 | Cook Island Maori |
| 60837 | 9T16.00 | Tongan |
| 55584 | 9T17.00 | Niuean |
| 25434 | 9T18.00 | Tokelauan |
| 64609 | 9T19.00 | Fijian |
| 46752 | 9T1A.00 | Other Pacific ethnic group |
| 46649 | 9T1B.00 | South East Asian |
| 12718 | 9T1C.00 | Chinese |
| 25920 | 9T1D.00 | Indian |
| 32396 | 9T1E.00 | Other Asian |
| 96789 | 9T1Y.00 | Other New Zealand ethnic group |
| 71425 | 9T1Z.00 | New Zealand ethnic group NOS |
| 32781 | 9T2..00 | Traveller - gypsy |
| 94487 | 9T3..00 | Yemeni |
| 12435 | 9i...00 | Ethnic category - 2001 census |
| 12351 | 9i0..00 | British or mixed British - ethnic category 2001 census |
| 98111 | 9i00.00 | White British - ethnic category 2001 census |
| 12532 | 9i1..00 | Irish - ethnic category 2001 census |
| 98213 | 9i10.00 | White Irish - ethnic category 2001 census |
| 12421 | 9i2..00 | Other White background - ethnic category 2001 census |
| 12352 | 9i20.00 | English - ethnic category 2001 census |
| 12436 | 9i21.00 | Scottish - ethnic category 2001 census |
| 12681 | 9i22.00 | Welsh - ethnic category 2001 census |
| 28887 | 9i23.00 | Cornish - ethnic category 2001 census |
| 42294 | 9i24.00 | Northern Irish - ethnic category 2001 census |
| 40102 | 9i25.00 | Ulster Scots - ethnic category 2001 census |
| 32778 | 9i26.00 | Cypriot (part not stated) - ethnic category 2001 census |
| 12355 | 9i27.00 | Greek - ethnic category 2001 census |
| 12769 | 9i28.00 | Greek Cypriot - ethnic category 2001 census |
| 12746 | 9i29.00 | Turkish - ethnic category 2001 census |
| 32413 | 9i2A.00 | Turkish Cypriot - ethnic category 2001 census |
| 12412 | 9i2B.00 | Italian - ethnic category 2001 census |
| 55223 | 9i2C.00 | Irish Traveller - ethnic category 2001 census |
| 55113 | 9i2D.00 | Traveller - ethnic category 2001 census |
| 42290 | 9i2E.00 | Gypsy/Romany - ethnic category 2001 census |
| 12467 | 9i2F.00 | Polish - ethnic category 2001 census |
| 12433 | 9i2G.00 | Baltic Estonian/Latvian/Lithuanian - ethn categ 2001 census |
| 28973 | 9i2H.00 | Commonwealth (Russian) Indep States - ethn categ 2001 census |
| 26341 | 9i2J.00 | Kosovan - ethnic category 2001 census |
| 25422 | 9i2K.00 | Albanian - ethnic category 2001 census |
| 46956 | 9i2L.00 | Bosnian - ethnic category 2001 census |
| 28866 | 9i2M.00 | Croatian - ethnic category 2001 census |
| 47074 | 9i2N.00 | Serbian - ethnic category 2001 census |
| 28936 | 9i2P.00 | Other republics former Yugoslavia - ethnic categ 2001 census |
| 26391 | 9i2Q.00 | Mixed Irish and other White - ethnic category 2001 census |
| 12402 | 9i2R.00 | Oth White European/European unsp/Mixed European 2001 census |
| 28900 | 9i2S.00 | Other mixed White - ethnic category 2001 census |
| 12591 | 9i2T.00 | Other White or White unspecified ethnic category 2001 census |
| 12742 | 9i3..00 | White and Black Caribbean - ethnic category 2001 census |
| 12437 | 9i4..00 | White and Black African - ethnic category 2001 census |
| 12638 | 9i5..00 | White and Asian - ethnic category 2001 census |
| 12873 | 9i6..00 | Other Mixed background - ethnic category 2001 census |
| 12795 | 9i60.00 | Black and Asian - ethnic category 2001 census |
| 49940 | 9i61.00 | Black and Chinese - ethnic category 2001 census |
| 40110 | 9i62.00 | Black and White - ethnic category 2001 census |
| 12706 | 9i63.00 | Chinese and White - ethnic category 2001 census |
| 47005 | 9i64.00 | Asian and Chinese - ethnic category 2001 census |
| 32408 | 9i65.00 | Other Mixed or Mixed unspecified ethnic category 2001 census |
| 12414 | 9i7..00 | Indian or British Indian - ethnic category 2001 census |
| 12460 | 9i8..00 | Pakistani or British Pakistani - ethnic category 2001 census |
| 28888 | 9i9..00 | Bangladeshi or British Bangladeshi - ethn categ 2001 census |
| 12513 | 9iA..00 | Other Asian background - ethnic category 2001 census |
| 26392 | 9iA1.00 | Punjabi - ethnic category 2001 census |
| 64133 | 9iA2.00 | Kashmiri - ethnic category 2001 census |
| 47077 | 9iA3.00 | East African Asian - ethnic category 2001 census |
| 12608 | 9iA4.00 | Sri Lankan - ethnic category 2001 census |
| 12760 | 9iA5.00 | Tamil - ethnic category 2001 census |
| 12887 | 9iA6.00 | Sinhalese - ethnic category 2001 census |
| 32399 | 9iA7.00 | Caribbean Asian - ethnic category 2001 census |
| 12653 | 9iA8.00 | British Asian - ethnic category 2001 census |
| 46056 | 9iA9.00 | Mixed Asian - ethnic category 2001 census |
| 28935 | 9iAA.00 | Other Asian or Asian unspecified ethnic category 2001 census |
| 12432 | 9iB..00 | Caribbean - ethnic category 2001 census |
| 12350 | 9iC..00 | African - ethnic category 2001 census |
| 32389 | 9iD..00 | Other Black background - ethnic category 2001 census |
| 12443 | 9iD0.00 | Somali - ethnic category 2001 census |
| 32886 | 9iD1.00 | Nigerian - ethnic category 2001 census |
| 40097 | 9iD2.00 | Black British - ethnic category 2001 census |
| 40096 | 9iD3.00 | Mixed Black - ethnic category 2001 census |
| 46047 | 9iD4.00 | Other Black or Black unspecified ethnic category 2001 census |
| 12468 | 9iE..00 | Chinese - ethnic category 2001 census |
| 12434 | 9iF..00 | Other - ethnic category 2001 census |
| 12719 | 9iF0.00 | Vietnamese - ethnic category 2001 census |
| 12473 | 9iF1.00 | Japanese - ethnic category 2001 census |
| 12420 | 9iF2.00 | Filipino - ethnic category 2001 census |
| 12730 | 9iF3.00 | Malaysian - ethnic category 2001 census |
| 63872 | 9iF4.00 | Buddhist - ethnic category 2001 census |
| 56127 | 9iF5.00 | Hindu - ethnic category 2001 census |
| 46063 | 9iF6.00 | Jewish - ethnic category 2001 census |
| 47091 | 9iF7.00 | Muslim - ethnic category 2001 census |
| 49658 | 9iF8.00 | Sikh - ethnic category 2001 census |
| 46059 | 9iF9.00 | Arab - ethnic category 2001 census |
| 47028 | 9iFA.00 | North African - ethnic category 2001 census |
| 28909 | 9iFB.00 | Mid East (excl Israeli, Iranian & Arab) - eth cat 2001 cens |
| 46964 | 9iFC.00 | Israeli - ethnic category 2001 census |
| 25937 | 9iFD.00 | Iranian - ethnic category 2001 census |
| 45964 | 9iFE.00 | Kurdish - ethnic category 2001 census |
| 25451 | 9iFF.00 | Moroccan - ethnic category 2001 census |
| 26246 | 9iFG.00 | Latin American - ethnic category 2001 census |
| 12756 | 9iFH.00 | South and Central American - ethnic category 2001 census |
| 32382 | 9iFJ.00 | Mauritian/Seychellois/Maldivian/St Helena eth cat 2001census |
| 26455 | 9iFK.00 | Any other group - ethnic category 2001 census |
| 12459 | 9iG..00 | Ethnic category not stated - 2001 census |

B) Deriving residence information from patient file in Hospital Episodes Statistics

| **Code** | **Description** | **Information extracted** |
| --- | --- | --- |
| 19 | The usual place of residence, including no fixed abode | Not used |
| 29 | Temporary place of residence when usually resident elsewhere, for example, hotels and residential educational establishments | Not used |
| 30 | Repatriation from high security psychiatric hospital (1999-00 to 2006-07) | residence: other |
| 37 | Penal establishment: court (1999-00 to 2006-07) | Not used |
| 38 | Penal establishment: police station (1999-00 to 2006-07) | Not used |
| 39 | Penal establishment (court and police station excluded from 1999-2000) | residence: other |
| 48 | High security psychiatric hospital, Scotland (1999-00 to 2006-07) | residence: other |
| 49 | NHS other hospital provider: high security psychiatric accommodation in an NHS hospital provider (NHS trust) | residence: other |
| 50 | NHS other hospital provider: medium secure unit (1999-00 to 2006-07) | residence: other |
| 51 | NHS other hospital provider: ward for general patients or the younger physically disabled or A&E department | Not used |
| 52 | NHS other hospital provider: ward for maternity patients or neonates | Not used |
| 53 | NHS other hospital provider: ward for patients who are mentally ill or have learning disabilities | Not used |
| 54 | NHS run nursing home, residential care home or group home | care home residence |
| 65 | Local authority Part 3 residential accommodation: where care is provided (from 1996-97) | sheltered accommodation |
| 66 | Local authority foster care, but not in Part 3 residential accommodation: where care is provided (from 1996-97) | residence: other |
| 69 | Local authority home or care (1989-90 to 1995-96) | care home residence |
| 79 | Babies born in or on the way to hospital | Not used |
| 85 | Non-NHS (other than Local Authority) run residential care home (from 1996-97) | care home residence |
| 86 | Non-NHS (other than Local Authority) run nursing home (from 1996-97 to 2006-07) | care home residence |
| 87 | Non-NHS run hospital | Not used |
| 88 | non-NHS (other than Local Authority) run hospice | residence: other |
| 89 | Non-NHS institution (1989-90 to 1995-96) | Not used |
| 98 | Not applicable | Not used |
| 99 | Not known | Not used |
