# Supplementary material for: Identifying social factors amongst older individuals in linked electronic health records: An assessment in a population based study
Source: PLoS One. 2017 Nov 30;12(11):e0189038. doi: 10.1371/journal.pone.0189038 (PMC5708811; doi:10.1371/journal.pone.0189038)
Supplement: S3 Table — (DOCX) [file pone.0189038.s003.docx]

S3 Table Pattern of completeness for social factors recording (N=591037(100%))

| **Social factors** | **Source: CPRD & HES**  Number of patients with complete information N (%) | **Source: CPRD, HES & family number**  Number of patients with complete information N (%) |
| --- | --- | --- |
| **All 8 social factors**  Living arrangements: living alone (yes/no)  Living arrangements: cohabitation (yes/no)  Marital status  Residence: place  Ethnicity  IMD  Immigration status  Religion | 45 (0.01%) | 53 (0.01%) |
| **6 social factors (excluding religion and immigration status)**  Living arrangements: living alone (yes/no)  Living arrangements: cohabitation (yes/no)  Marital status  Residence: place  Ethnicity  IMD | 13042 (2.2%) | 22477 (3.8%) |
| **5 social factors (additionally excluding residence)**  Living arrangements: living alone (yes/no)  Living arrangements: cohabitation (yes/no)  Marital status  Ethnicity  IMD | 81583 (13.8%) | 222600 (37.7%) |
| **4 social factors (additionally excluding marital status)**  Living arrangements: living alone (yes/no)  Living arrangements: cohabitation (yes/no)  Ethnicity  IMD | 84974 (14.4%) | 246609 (41.7%) |
| **3 social factors (additionally excluding cohabitation)**  Living arrangements: living alone (yes/no)  Ethnicity  IMD | 123450 (20.9%) | 290912 (49.2%) |

CPRD Clinical Practice Research Datalink HES Hospital Episodes Statistics IMD index of multiple deprivation
