# Supplementary material for: Identifying social factors amongst older individuals in linked electronic health records: An assessment in a population based study
Source: PLoS One. 2017 Nov 30;12(11):e0189038. doi: 10.1371/journal.pone.0189038 (PMC5708811; doi:10.1371/journal.pone.0189038)
Supplement: S4 Table — (DOCX) [file pone.0189038.s004.docx]

**S4 Table Time varying social factors and data source: discordant information on same date and missing event dates (total study population 591037(100%))**

| **Social factors and source of their information** | | **Number of patients (%) with information on social factor prior to dropping discordant information on same date** | **Number of patients (%) excluded due to discordant information on same date** | **Number of patients (%) with information on social factor after dropping discordant information on same date** | **Missing event date replaced with system date#**  **Number of patients (%)** |
| --- | --- | --- | --- | --- | --- |
| Living arrangements | Living alone (yes/no) (CPRD & HES) | 173046 (29.3%) | 456 (0.1%) | 172590 (29.2%) | 1812 (0.3%) |
|  | Living alone (yes/no) (CPRD only) | 172085 (29.1%) | 460 (0.1%) | 171625 (29%) | 1812 (0.3%) |
|  | Living alone (yes/no) (CPRD, HES & FN) | 413694 (70%) | 456 (0.1%) | 413238 (69.9%) | 1812 (0.3%) |
|  | Cohabitation (yes/no) (CPRD & HES) | 128641 (21.8%) | 68 (0.01%) | 128573 (21.8%) | 1916 (0.3%) |
|  | Cohabitation (yes/no) (CPRD only) | 128641 (21.8%) | 68 (0.01%) | 128573 (21.8%) | 1916 (0.3%) |
|  | Cohabitation (yes/no) (CPRD, HES & FN) | 356870 (60.4%) | 68 (0.01%) | 356802 (60.4%) | 1916 (0.3%) |
| Marital status | Marital status (CPRD only) | 160963 (27.2%) | 151 (0.03%) | 160812 (27.2%) | 2061 (0.3%) |
|  | Marital status (CPRD, HES & FN) | 351432 (59.5%) | 151 (0.03%) | 351281 (59.4%) | 2061 (0.3%) |
| Residence | Residence: place (CPRD & HES) | 60811 (10.3%) | 173 (0.03%) | 60638 (10.3%) | 61 (0.01%) |
|  | Residence: place (CPRD only) | 59437 (10.1%) | 174 (0.03%) | 59263 (10%) | 61 (0.01%) |
|  | Residence: place (CPRD, HES & FN)~ | 65140 (11%) | 173 (0.03%) | 64967 (11%) | 61 (0.01%) |
|  | Residence: place (CPRD, HES & FN)* | 64876 (11%) | 173 (0.03%) | 64703 (10.9%) | 61 (0.01%) |
|  | Residence: homelessness (CPRD & HES) | 60813 (10.3%) | 4 (<0.001%) | 60809 (10.3%) | 64 (0.01%) |
|  | Residence: homelessness (CPRD) | 59439 (10.1%) | 4 (<0.001%) | 59435 (10.1%) | 64 (0.01%) |

CPRD Clinical Practice Research Datalink HES Hospital Episodes Statistics FN Family number

# Overall event date was missing for 2219 (0.4%) patients

~ 1^st^ criteria for care home residence using family number: households with ≥3 individuals aged ≥65 years and were in majority compared to those <65 years of age

*2nd criteria for care home residence using family number: households with ≥3 individuals aged ≥65 years, ≤3 individuals aged ≤50 years and those aged ≥65 years were in majority
