# Supplementary material for: Identifying social factors amongst older individuals in linked electronic health records: An assessment in a population based study
Source: PLoS One. 2017 Nov 30;12(11):e0189038. doi: 10.1371/journal.pone.0189038 (PMC5708811; doi:10.1371/journal.pone.0189038)
Supplement: S5 Table — (DOCX) [file pone.0189038.s005.docx]

**S5 Table Additional information obtained from using family number**

| Social factors | | Information from CPRD, HES & family number (number of patients with data available)  Total study population 591037 (100%) | | Information from CPRD,& HES  Total study population 591037(100%) | |
| --- | --- | --- | --- | --- | --- |
|  |  | Frequency (%) | Missing data | Frequency (%) | Missing data |
| Living arrangements: living alone | No | 407544 (69%) | 177799 (30.1%) | 166896 (28.2%) | 418447 (70.8%) |
|  | Yes | 5694 (1%) |  | 5694 (1%) |  |
| Living arrangements: Cohabitation | No | 48445 (8.2%) | 234235 (39.6%) | 14666 (2.5%) | 462464 (78.2%) |
|  | Yes | 308357 (52.2%) |  | 113907 (19.3%) |  |
| Residence: place | Care home | 33205 (5.6%) | 526070 (89%) | 28876 (4.9%) | 530399 (89.7%) |
|  | Sheltered | 1272 (0.2%) |  | 1272 (0.2%) |  |
|  | Household | 29296 (5%) |  | 29296 (5%) |  |
|  | Others | 1194 (0.2%) |  | 1194 (0.2%) |  |
| Marital status | Single | 7291 (1.2%) | 239756 (40.6%) | 7291 (1.2%) | 430225 (72.8%) |
|  | Married/Civil | 108921 (18.4%) |  | 108921 (18.4%) |  |
|  | Widow/er | 30459 (5.2%) |  | 30459 (5.1%) |  |
|  | Divorced | 7446 (1.3%) |  | 7446 (1.3%) |  |
|  | Separated | 2100 (0.3%) |  | 2100 (0.4%) |  |
|  | Partner uncategorised/other^#^ | 195064 (33%) |  | 4595 (0.8%) |  |

^#^Due to very small numbers in `Partner: other’ category the data are presented with `Partner: uncategorised’ CPRD Clinical Practice Research Datalink HES Hospital Episodes Statistics
