# Supplementary material for: Identifying social factors amongst older individuals in linked electronic health records: An assessment in a population based study
Source: PLoS One. 2017 Nov 30;12(11):e0189038. doi: 10.1371/journal.pone.0189038 (PMC5708811; doi:10.1371/journal.pone.0189038)
Supplement: S2 Fig — (DOCX) [file pone.0189038.s007.docx]

**S2 Fig Timeliness of recording of cohabitation and marital status: comparing data from Clinical Practice Research Datalink (CPRD) and Hospital Episodes Statistics (HES) with data obtained from CPRD, HES and family number**


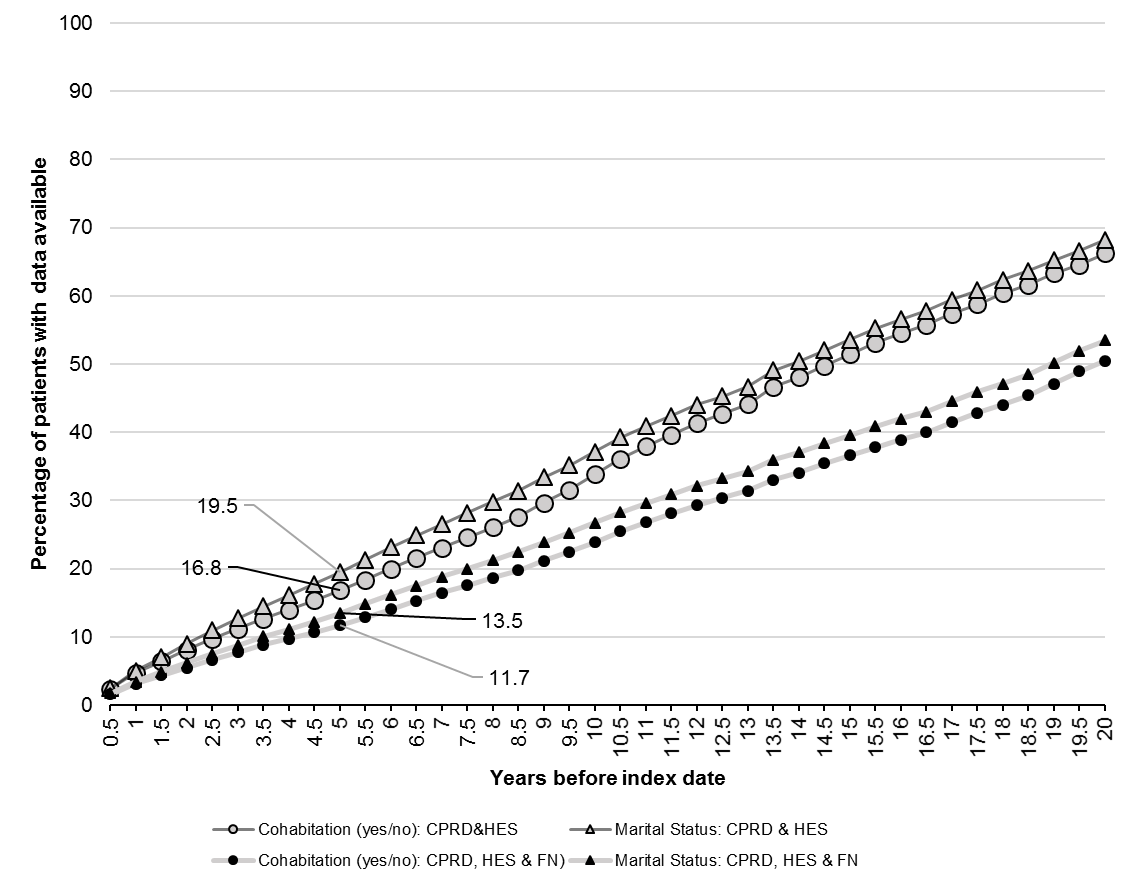


CPRD Clinical Practice Research Datalink HES Hospital Episodes Statistics FN family number Index date=01/01/2013
