# Supplementary material for: Identifying social factors amongst older individuals in linked electronic health records: An assessment in a population based study
Source: PLoS One. 2017 Nov 30;12(11):e0189038. doi: 10.1371/journal.pone.0189038 (PMC5708811; doi:10.1371/journal.pone.0189038)
Supplement: S3 Fig — (DOCX) [file pone.0189038.s008.docx]

**S3 Fig Proportion of total study population (n=591037) with recording of time varying social factors within 5 years of index date (01/01/2013)**

CPRD Clinical Practice Research Datalink HES Hospital Episodes Statistics FN family number
